# Supplementary material for: Out of the blue: family‐wide loss of anthocyanin biosynthesis in Cucurbitaceae
Source: Plant J. 2026 Sep 24;127(6):e71136. doi: 10.1111/tpj.71136 (PMC13602278; doi:10.1111/tpj.71136)
Supplement: Supplementary file 1 [file TPJ-127-0-s001.pdf]

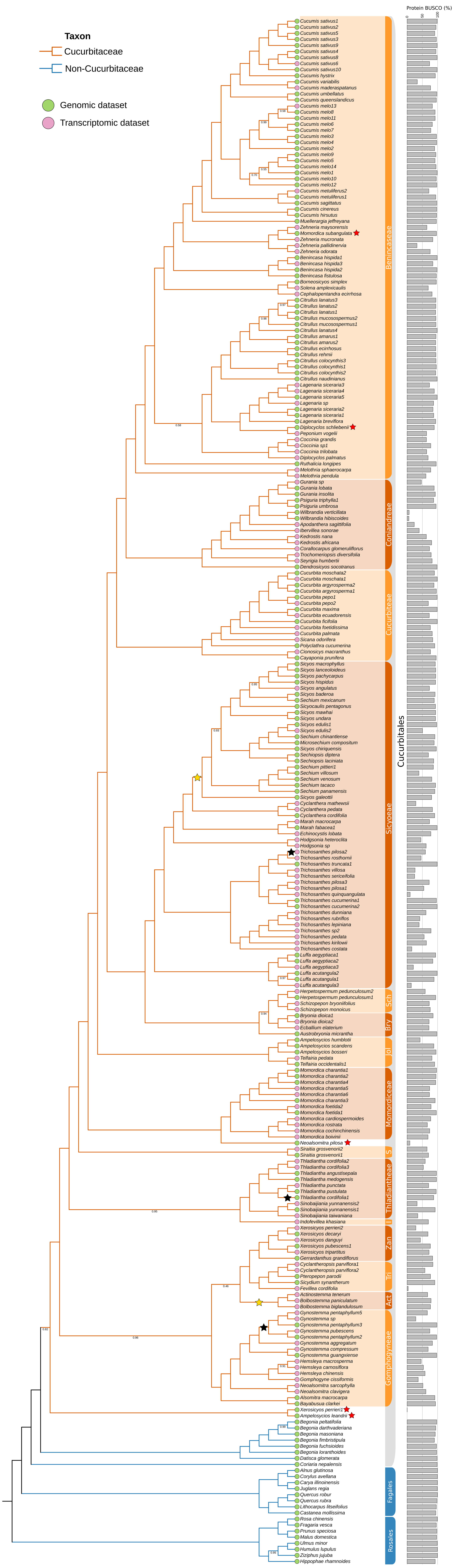

Fig. S1: Coalescence species tree constructed from 2805 BUSCO orthogroups across 255 non-contaminated datasets.
Numbers on branches represent local posterior probability if below 1. The 15 Cucurbitaceae tribes are highlighted with orange coloured backgrounds. Branches corresponding to Cucurbitaceae are shown in orange, while non-Cucurbitaceae outgroups are shown in blue. Dataset type (genomic or transcriptomic) is indicated at the terminal tips in green and pink, respectively. The percentage of conserved BUSCO genes identified in the polypeptide sequences of each dataset is shown on the far right. Species names follow Plants of the World Online (POWO; powo.science.kew.org). Datasets excluded from the subsequent trees due to suspicious or inconclusive placements are marked with red stars. *Xerosicyos perrieri*1 and *Ampelosycios leandrii* were removed due to extremely low completeness (0.1% and 0% BUSCO, respectively). *Neoalsomitra pilosa* (9% BUSCO) and *Momordica subangulata* (96.4% BUSCO) clustered outside their expected tribes, likely reflecting low completeness or misidentified material. *Diplocyclos schliebenii* grouped with *Peponium* intead of *Coccinia* suggesting misidentification. Species/clades marked with black stars are retained in subsequent trees but warrants caution. For example, multiple accessions of *Gynostemma pentaphyllum* did not form a monophyletic clade; instead, *G. pubescens* was nested among them, which may suggest incomplete lineage sorting, introgression between the two species, or potential mis-identification. Similary, accession of *Thladiantha cordifolia* (1,2,3) and *Trichosanthes pilosa* (1,2,3) failed to form monophyletic groups. The species in the clade marked with yellow stars were merged into a single genus based on nested phylogenetic positions (Schaefer & Renner, 2011): *Bolbostemma* in *Actinostemma*; *Microsechium, Sicyocaulis, Sicyosperma, Sechiopsis*, and *Sechium* were all subsumed within *Sicyos*. The clustering of *C. lanatus* with *C. mucosospermus* is consistent with previous phylogenetic studies (Chomicki & Renner, 2015). Tribe abbreviations: Act, Actinostemmateae; Tri, Triceratieae; Zan, Zanonieae; I, Indofevilleae; S, Siraitieae; Jol, Joliffieae; Bry, Bryonieae; Sch; Schizopeponeae

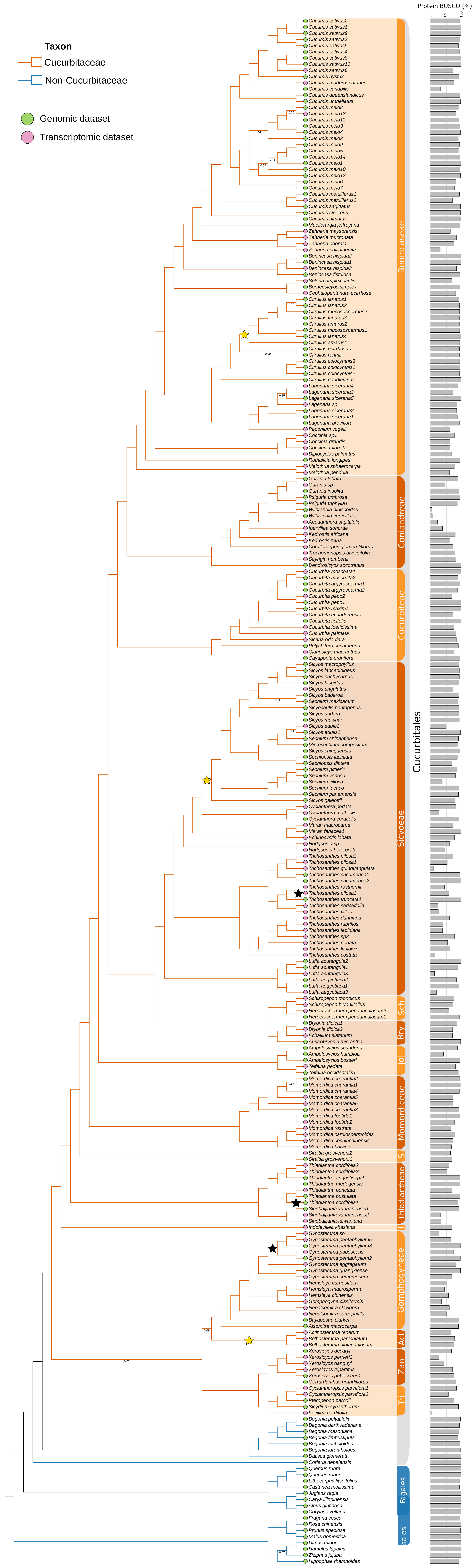

Fig. S2: Coalescence species tree constructed from 2805 BUSCO orthogroups across 250 non-contaminated datasets.

Numbers on branches represent local posterior probability if below 1. The 15 Cucurbitaceae tribes are highlighted with orange coloured backgrounds. Branches corresponding to Cucurbitaceae are shown in orange, while non-Cucurbitaceae outgroups are shown in blue. Dataset type (genomic or transcriptomic) is indicated at the terminal tips in green and pink, respectively. The percentage of conserved BUSCO genes identified in the polypeptide sequences of each dataset is shown on the far right. Species names follow Plants of the World Online (POWO; powo.science.kew.org). Species/clades marked with black stars are retained but warrants caution. For example, multiple accessions of *Gynostemma pentaphyllum* did not form a monophyletic clade; instead, *G. pubescens* was nested among them, which may suggest incomplete lineage sorting, introgression between the two species, or potential misidentification. Similary, accession of *Thladiantha cordifolia* (1,2,3) and *Trichosanthes pilosa* (1,2,3) failed to form monophyletic groups. The species in the clade marked with yellow stars were merged into a single genus based on nested phylogenetic positions (Schaefer and Renner, 2011): *Bolbostemma* in *Actinostemma*; *Microsechium*, *Sicyocaulis*, *Sicyosperma*, *Sechiopsis*, and *Sechium* were all subsumed within *Sicyos*. The clustering of *C. lanatus* with *C. mucosospermus* is consistent with previous phylogenetic studies (Chomicki & Renner, 2015). Tribe abbreviations: Act, Actinostemmateae; Tri, Triceratieae; Zan, Zanonieae; I; Indofevilleae; S, Siraitieae; Jol, Joliffieae; Bry, Bryonieae; Sch; Schizopeponeae

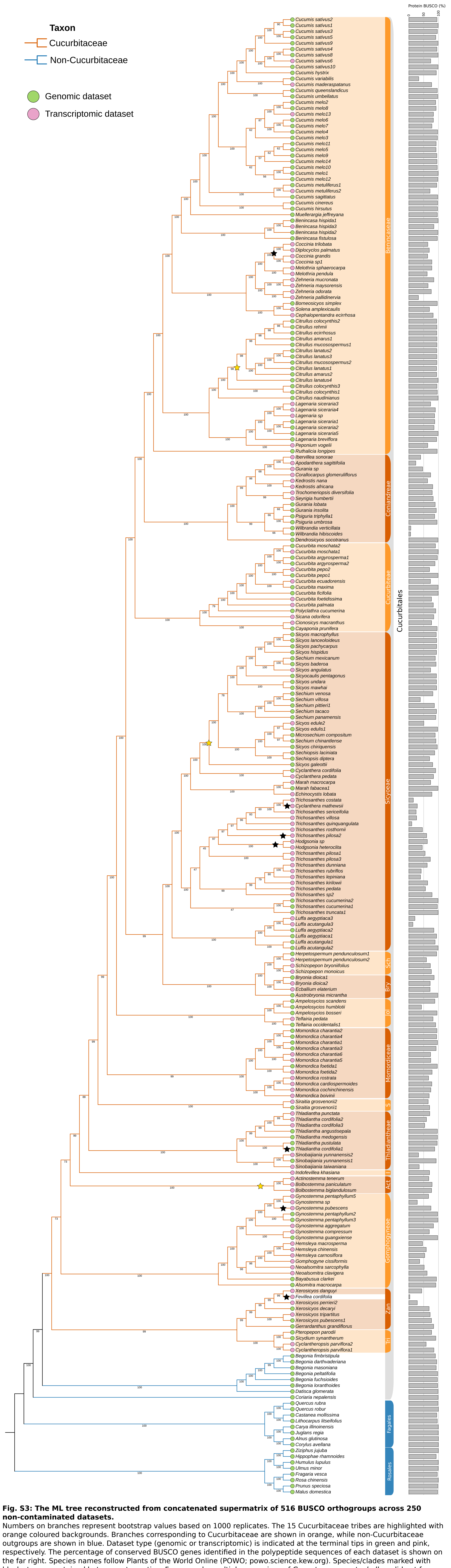

Fig. S3: The ML tree reconstructed from concatenated supermatrix of 516 BUSCO orthogroups across 250 non-contaminated datasets.
Numbers on branches represent bootstrap values based on 1000 replicates. The 15 Cucurbitaceae tribes are highlighted with orange coloured backgrounds. Branches corresponding to Cucurbitaceae are shown in orange, while non-Cucurbitaceae outgroups are shown in blue. Dataset type (genomic or transcriptomic) is indicated at the terminal tips in green and pink, respectively. The percentage of conserved BUSCO genes identified in the polypeptide sequences of each dataset is shown on the far right. Species names follow Plants of the World Online (POWO; powo.science.kew.org). Species/clades marked with black stars are retained but warrants caution. For example, multiple accessions of *Gynostemma pentaphyllum* did not form a monophyletic clade; instead, *G. pubescens* was nested among them, which may suggest incomplete lineage sorting, introgression between the two species, or potential mis-identification. Similary, accession of *Thladiantha cordifolia* (1,2,3) and *Trichosanthes pilosa* (1,2,3) failed to form monophyletic groups. *Hodgsonia* is nested between *Trichosanthes*. *Diplocyclos palmatus* is nested between *Coccinia*. *Fevillea cordifolia* (3.5% BUSCO) from the Triceratieae tribe is clustered in the Zanonieae tribe. The species in the clade marked with yellow stars were merged into a single genus based on nested phylogenetic positions (Schaefer and Renner, 2011): *Bolbostemma* in *Actinostemma*; *Microsechium, Sicyocaulis, Sicyosperma, Sechiopsis*, and *Sechium* were all subsumed within *Sicyos*. The clustering of *C. lanatus* with *C. mucosospermus* is consistent with previous phylogenetic studies (Chomicki & Renner, 2015). Tribe abbreviations: Act, Actinostemmateae; Tri, Triceratieae; Zan, Zanonieae; I, Indofevilleae; S, Siraitieae; Jol, Joliffieae; Bry, Bryonieae; Sch; Schizopeponeae

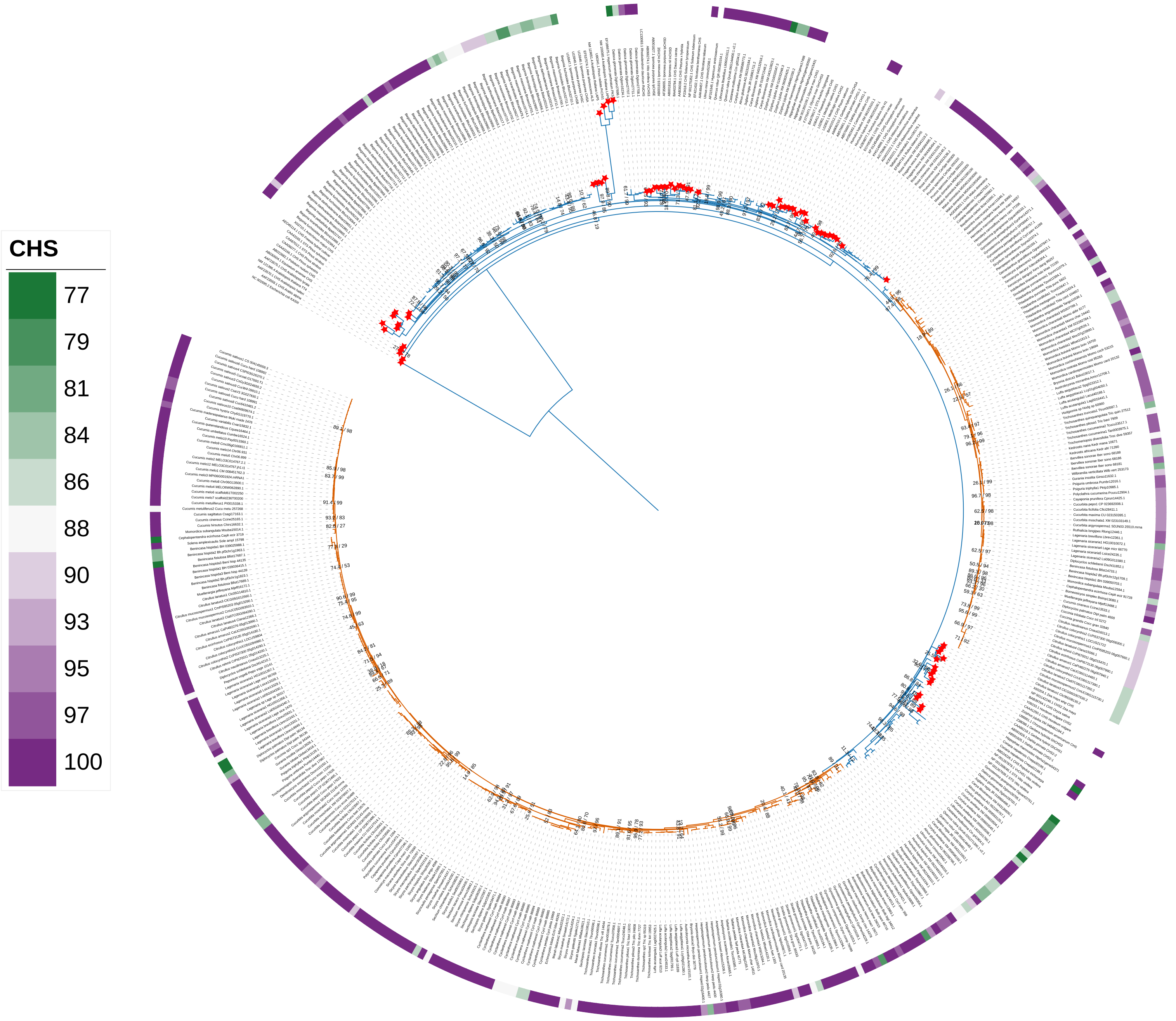

Fig. S4a: Codon-based maximum-likelihood phylogenetic tree of CHS sequences constructed with MAFFT and IQ-TREE.

The best-fit substitution model selected by ModelFinder was SYM+R7. Cucurbitaceae sequences are shown in orange branches, and non-Cucurbitaceae sequences in blue. Red stars at the tips of the terminal branches indicate functional enzymatic sequences identified in previous studies. Numbers above nodes represent Shimodaira-Hasegawa approximate likelihood ratio test (SH-aLRT) support (%) / ultrafast bootstrap support (%) based on 1000 replicates. Only percentages below 100 are shown. The outermost colour gradient indicates the percentage of conserved functionally important residues for the corresponding function, thereby reflecting the degree of functional conservation of each sequence. Polyketide synthase III sequences are used as outgroups. Abbreviations: CHS, Chalcone synthase; KAS III, Ketoacyl-ACP synthase III; LAP, Less adhesive pollen; PKS, polyketide synthase; ALS, Aloesone synthase; STS, Stilbene synthase

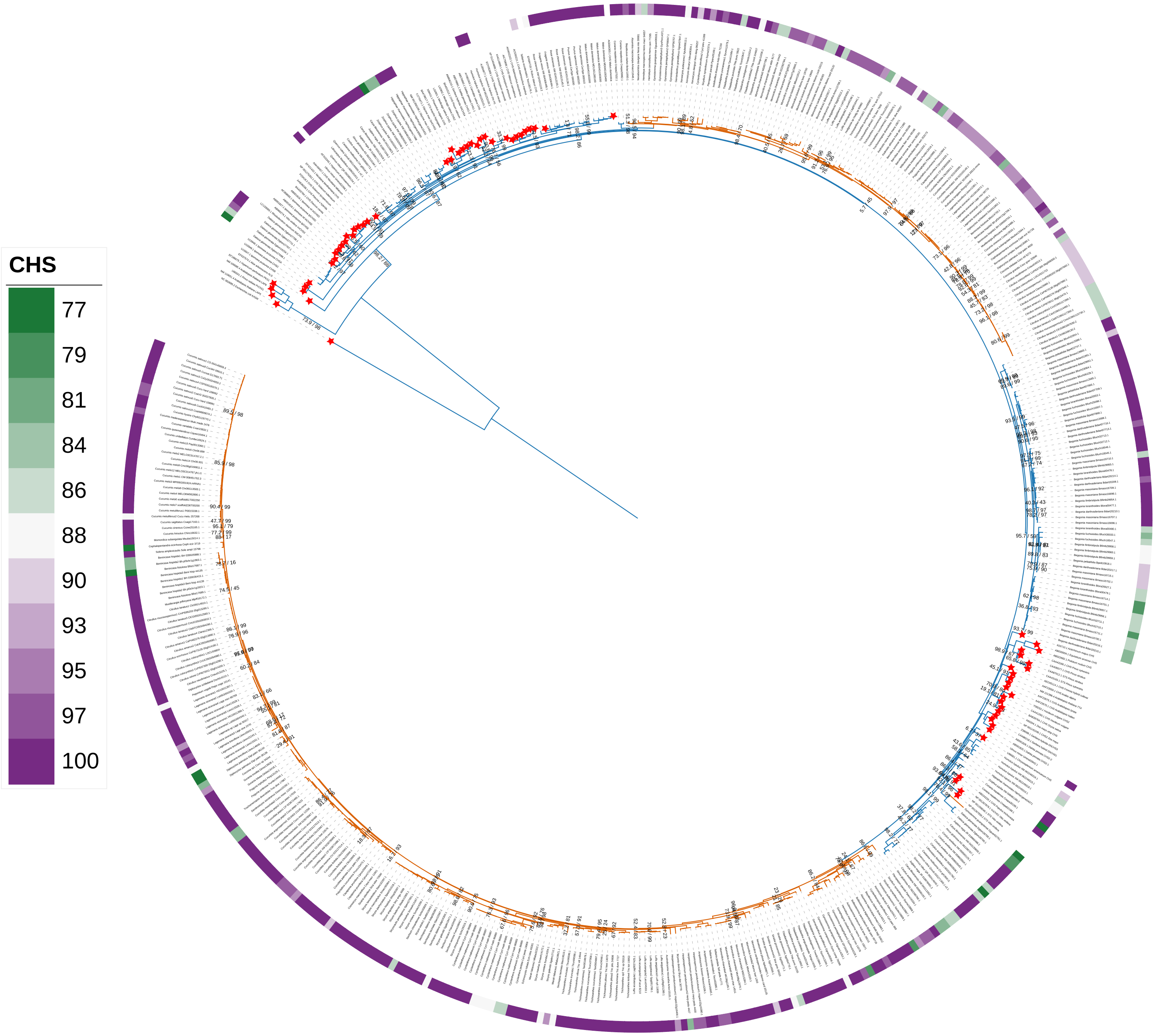

Fig. S4b: Codon-based maximum-likelihood phylogenetic tree of CHS sequences constructed with MUSCLE and IQ-TREE.
The best-fit substitution model selected by ModelFinder was SYM+R7. Cucurbitaceae sequences are shown in orange branches, and non-Cucurbitaceae sequences in blue. Red stars at the tips of the terminal branches indicate functional enzymatic sequences identified in previous studies. Numbers above nodes represent Shimodaira-Hasegawa approximate likelihood ratio test (SH-aLRT) support (%) / ultrafast bootstrap support (%) based on 1000 replicates. Only percentages below 100 are shown. The outermost colour gradient indicates the percentage of conserved functionally important residues for the corresponding function, thereby reflecting the degree of functional conservation of each sequence. Polyketide synthase III sequences are used as outgroups. Abbreviations: CHS, Chalcone synthase; KAS III, Ketoacyl-ACP synthase III; LAP, Less adhesive pollen; PKS, polyketide synthase; ALS, Aloesone synthase; STS, Stilbene synthase

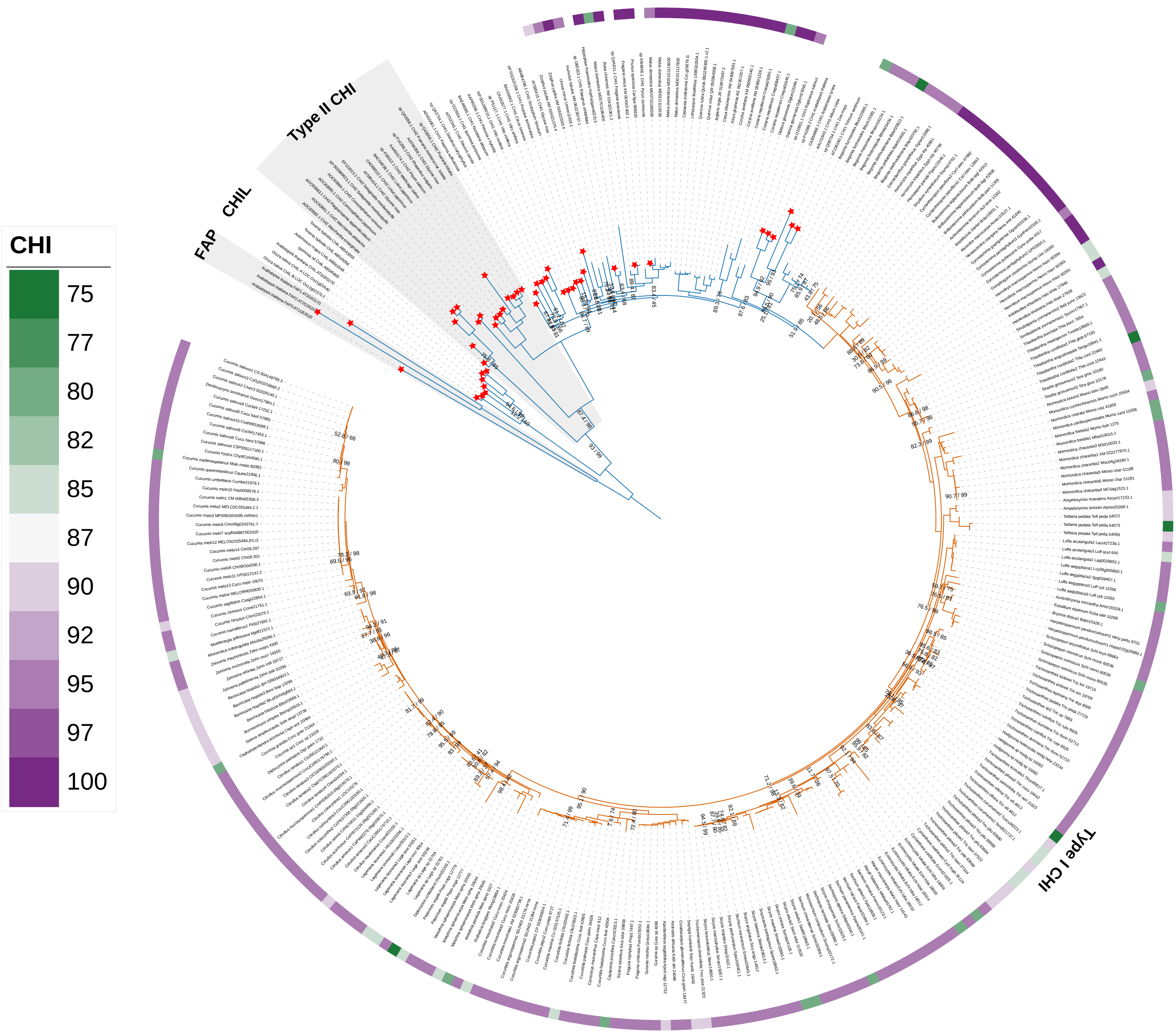

Fig. S5a: Codon-based maximum-likelihood phylogenetic tree of CHI sequences constructed with MAFFT and IQ-TREE.
The best-fit substitution model selected by ModelFinder was GTR+F+R6. Cucurbitaceae sequences are shown in orange branches, and non-Cucurbitaceae sequences in blue. Red stars at the tips of terminal branches indicate functional enzymatic sequences identified in previous studies. Numbers above nodes represent Shimodaira-Hasegawa approximate likelihood ratio test (SH-aLRT) support (%)/ ultrafast bootstrap support (%) based on 1000 replicates. Only percentages below 100 are shown. The outermost colour gradient indicates the percentage of conserved functionally important residues for the corresponding function, thereby reflecting the degree of functional conservation of each sequence.Abbreviations: FAP, Fatty acid binding protein; CHIL, Chalcone isomerase-like; CHI, Chalcone isomerase

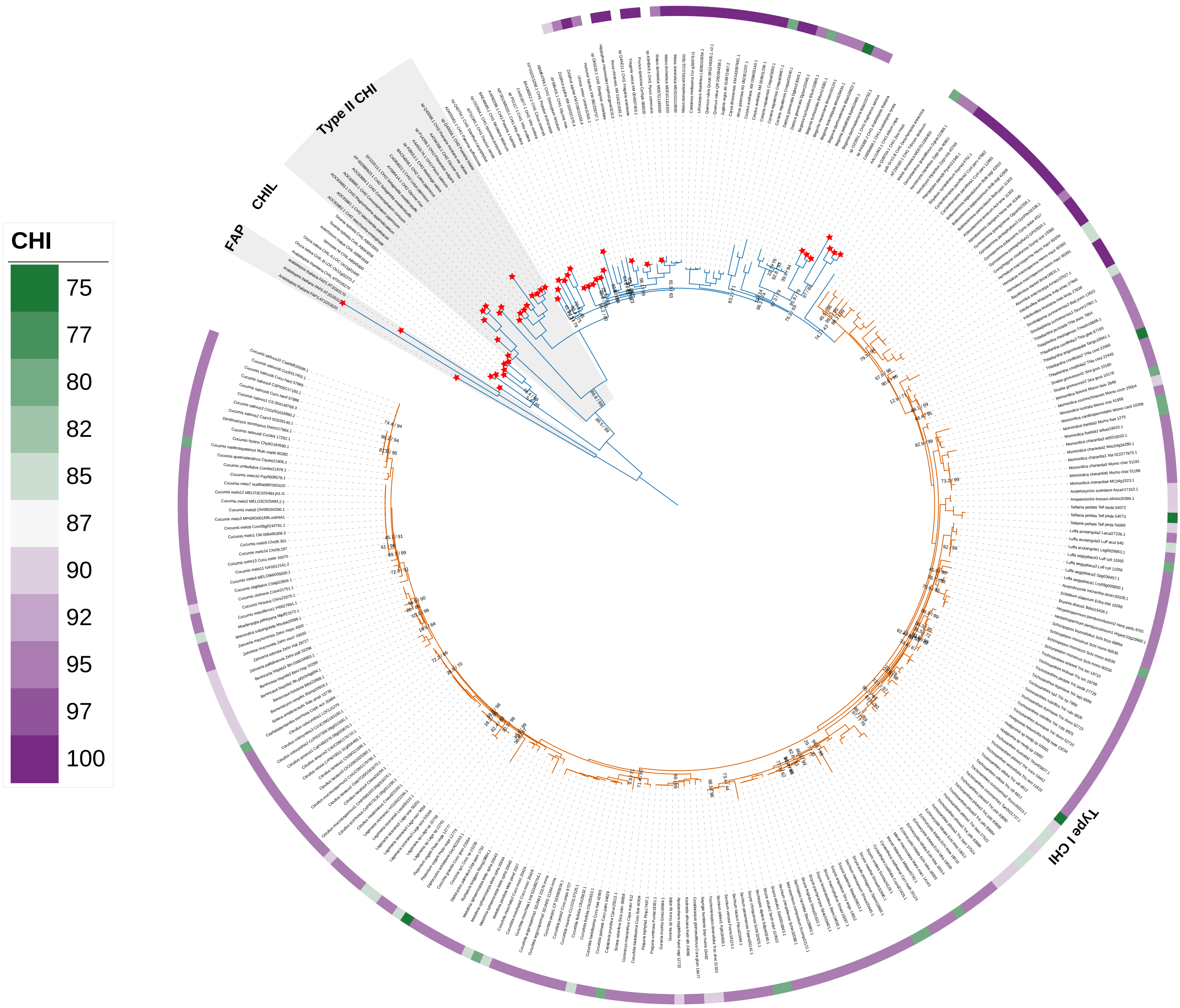

Fig. S5b: Codon-based maximum-likelihood phylogenetic tree of CHI sequences constructed with MUSCLE and IQ-TREE.

The best-fit substitution model selected by ModelFinder was GTR+F+R6. Cucurbitaceae sequences are shown in orange branches, and non-Cucurbitaceae sequences in blue. Red stars at the tips of terminal branches indicate functional enzymatic sequences identified in previous studies. Numbers above nodes represent Shimodaira-Hasegawa approximate likelihood ratio test (SH-aLRT) support (%)/ ultrafast bootstrap support (%) based on 1000 replicates. Only percentages below 100 are shown. The outermost colour gradient indicates the percentage of conserved functionally important residues for the corresponding function, thereby reflecting the degree of functional conservation of each sequence. Abbreviations: FAP, Fatty acid binding protein; CHIL, Chalcone isomerase-like; CHI,Chalcone isomerase

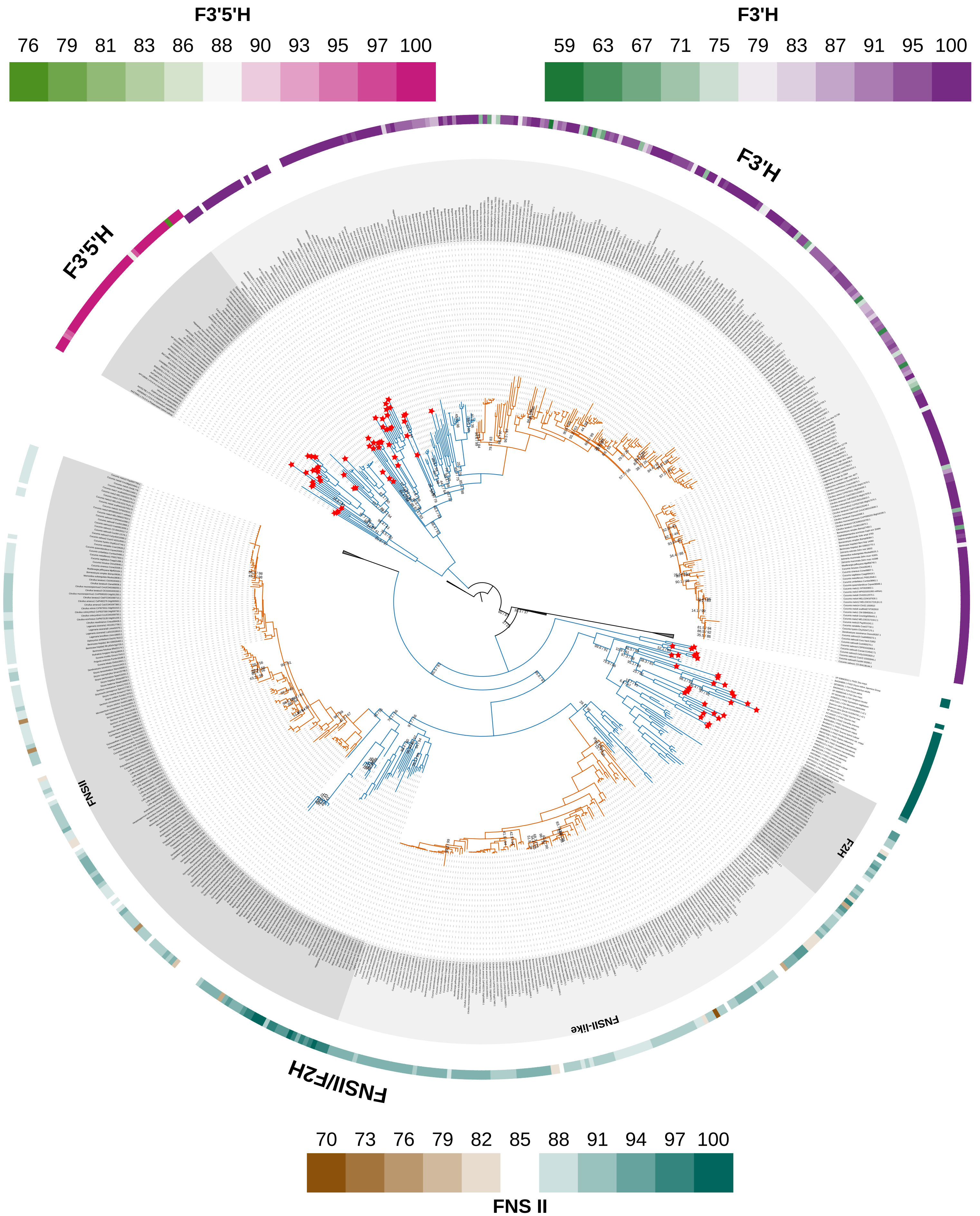

Fig. S6a: Codon-based maximum-likelihood phylogenetic tree of CYP450 sequences constructed with MAFFT and IQ-TREE.

The best-fit substitution model selected by Model-Finder was GTR+F+R10. Cucurbitaceae sequences are shown in orange branches, and non-Cucurbitaceae sequences shown in blue. Red stars at the tips of terminal branches indicate functional enzymatic sequences identified in previous studies. Numbers above nodes represent Shimodaira-Hasegawa approximate likelihood ratio test (SH-aLRT) support (%)/ ultrafast bootstrap support (%) based on 1000 replicates. Only percentages below 100 are shown. The outermost colour gradient indicates the percentage of conserved functionally important residues for the corresponding function, thereby reflecting the degree of functional conservation of each sequence. F3'H, flavonoid 3'-hydroxylase; F3'5'H, flavonoid 3',5'-hydroxylase; F2H, flavanone 2-hydroxylase; FNSII, flavone synthase II

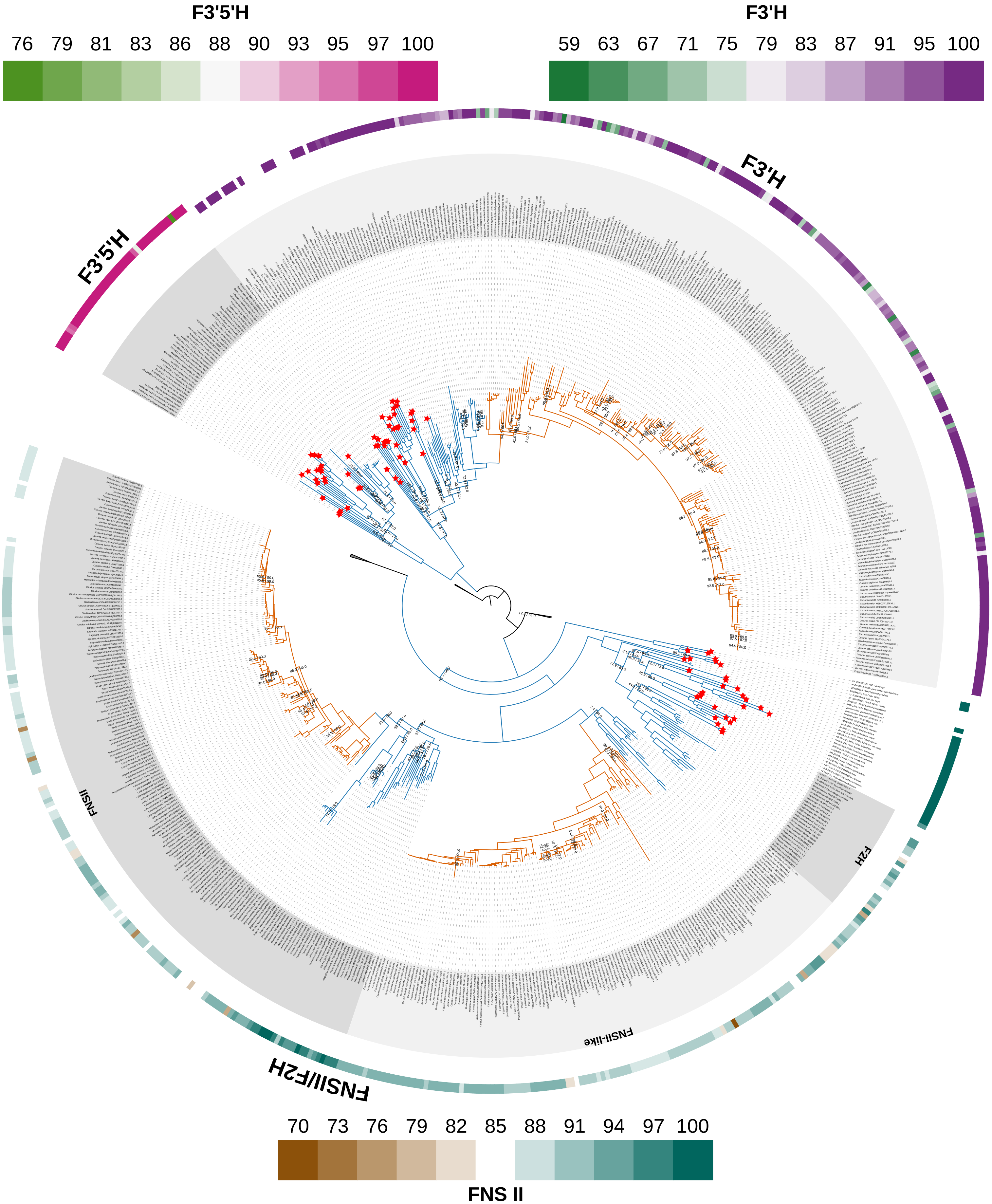

Fig. S6b: Codon-based maximum-likelihood phylogenetic tree of CYP450 sequences constructed with MUSCLE and IQ-TREE.

The best-fit substitution model selected by Model-Finder was GTR+F+I+R10. Cucurbitaceae sequences are shown in orange branches, and non-Cucurbitaceae sequences shown in blue. Red stars at the tips of terminal branches indicate functional enzymatic sequences identified in previous studies. Numbers above nodes represent Shimodaira-Hasegawa approximate likelihood ratio test (SH-aLRT) support (%)/ ultrafast bootstrap support (%) based on 1000 replicates. Only percentages below 100 are shown. The outermost colour gradient indicates the percentage of conserved functionally important residues for the corresponding function, thereby reflecting the degree of functional conservation of each sequence. F3'H, flavonoid 3'-hydroxylase; F3'5'H, flavonoid 3',5'-hydroxylase; F2H, flavanone 2-hydroxylase; FNSII, flavone synthase II

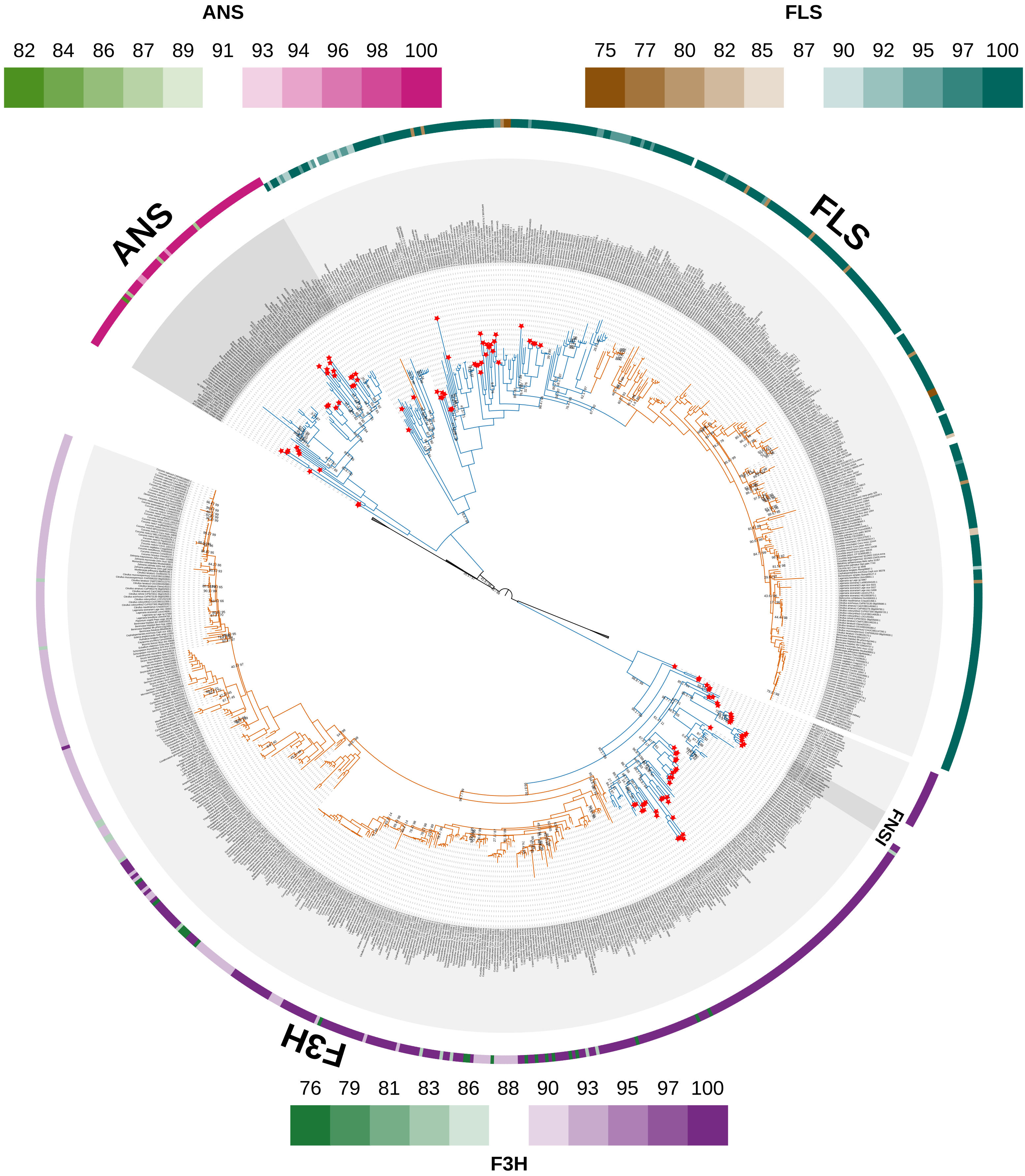

Fig. S7a: Codon-based maximum-likelihood phylogenetic tree of 2ODD sequences constructed with MAFFT and IQ-TREE.

The best-fit substitution model selected by Model-Finder was GTR+F+I+R9. Cucurbitaceae sequences are shown in orange branches, and non-Cucurbitaceae sequences shown in blue. Red stars at the tip of terminal branches indicate functional enzymatic sequences identified in previous studies. Numbers above nodes represent Shimodaira-Hasegawa approximate likelihood ratio test (SH-aLRT) support (%) / ultrafast bootstrap support (%) based on 1000 replicates. Only percentages below 100 are shown. The outermost colour gradient indicates the percentage of conserved functionally important residues for the corresponding function, thereby reflecting the degree of functional conservation of each sequence. ANS, anthocyanidin synthase; FLS, flavonol synthase; FNSI, flavone synthase I; F3H, flavanone-3-hydroxylase

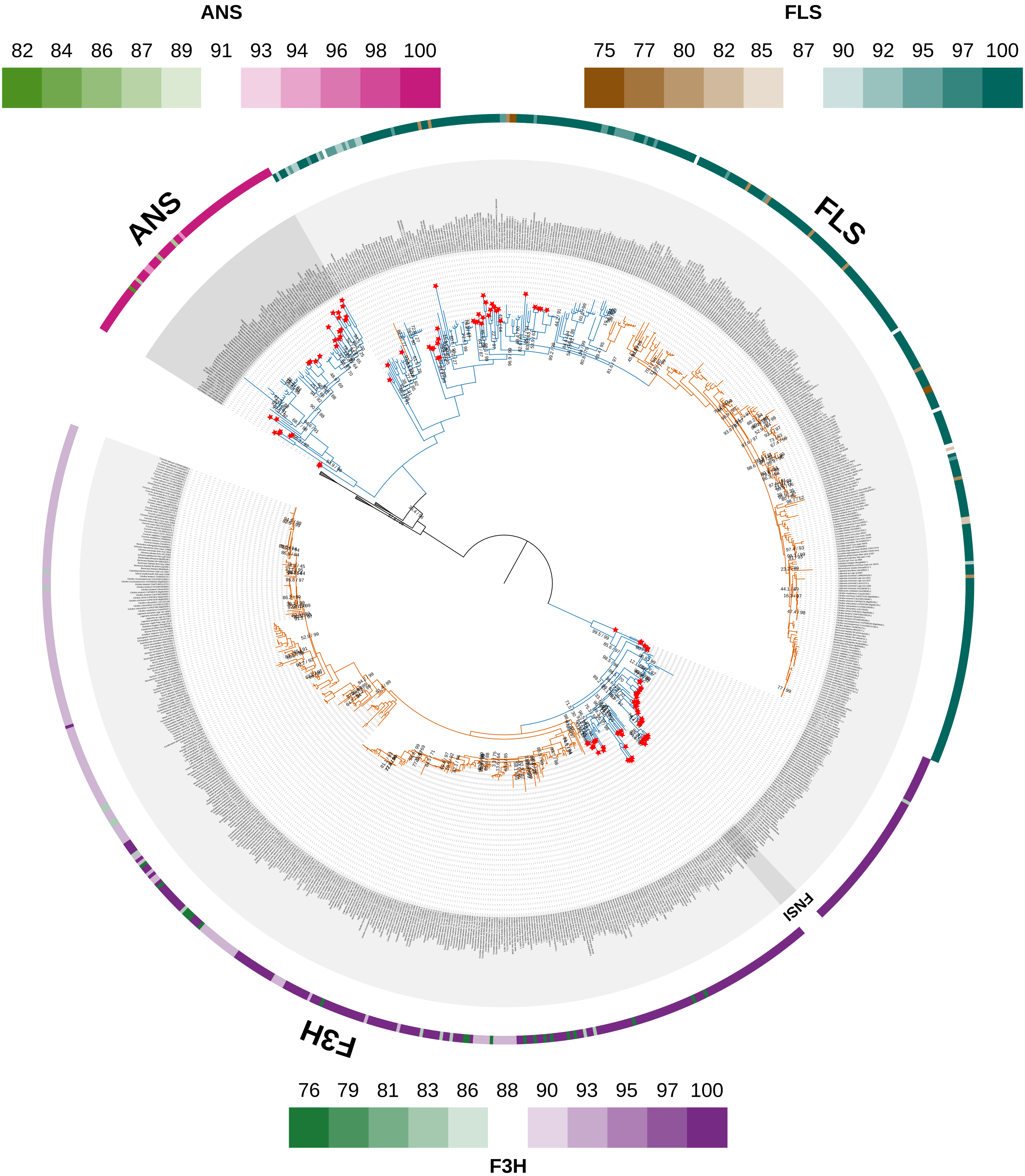

Fig. S7b: Codon-based maximum-likelihood phylogenetic tree of 2ODD sequences constructed with MUSCLE and IQ-TREE.

The best-fit substitution model selected by Model-Finder was GTR+F+I+R9. Cucurbitaceae sequences are shown in orange branches, and non-Cucurbitaceae sequences shown in blue. Red stars at the tip of terminal branches indicate functional enzymatic sequences identified in previous studies. Numbers above nodes represent Shimodaira-Hasegawa approximate likelihood ratio test (SH-aLRT) support (%) / ultrafast bootstrap support (%) based on 1000 replicates. Only percentages below 100 are shown. The outermost colour gradient indicates the percentage of conserved functionally important residues for the corresponding function, thereby reflecting the degree of functional conservation of each sequence. ANS, anthocyanidin synthase; FLS, flavonol synthase; FNSI, flavone synthase I; F3H, flavanone-3-hydroxylase

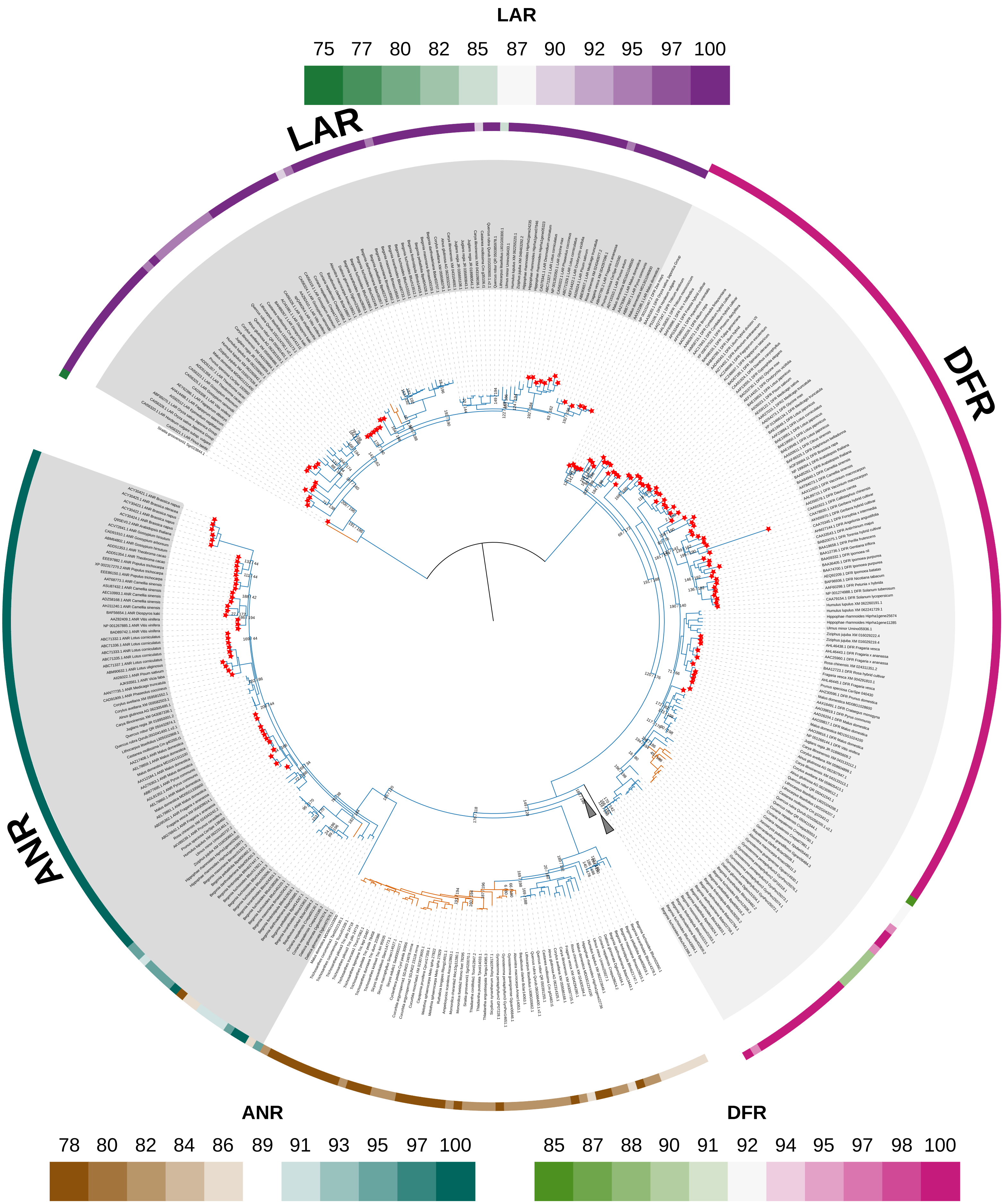

Fig. S8a: Codon-based maximum-likelihood phylogenetic tree of SDR sequences constructed with MAFFT and IQ-TREE.
The best-fit substitution model selected by Model-Finder was GTR+F+R10. Cucurbitaceae sequences are shown in orange branches, and non-Cucurbitaceae sequences shown in blue. Red stars at the tip of terminal branches indicate functional enzymatic sequences identified in previous studies. Numbers above nodes represent Shimodaira-Hasegawa approximate likelihood ratio test (SH-aLRT) support (%)/ ultrafast bootstrap support (%) based on 1000 replicates. Only percentages below 100 are shown. The outermost colour gradient indicates the percentage of conserved functionally important residues for the corresponding function, thereby reflecting the degree of functional conservation of each sequence. LAR, leucoanthocyanidin reductase; DFR, dihydroflavonol reductase; ANR, anthocyanidin reductase.

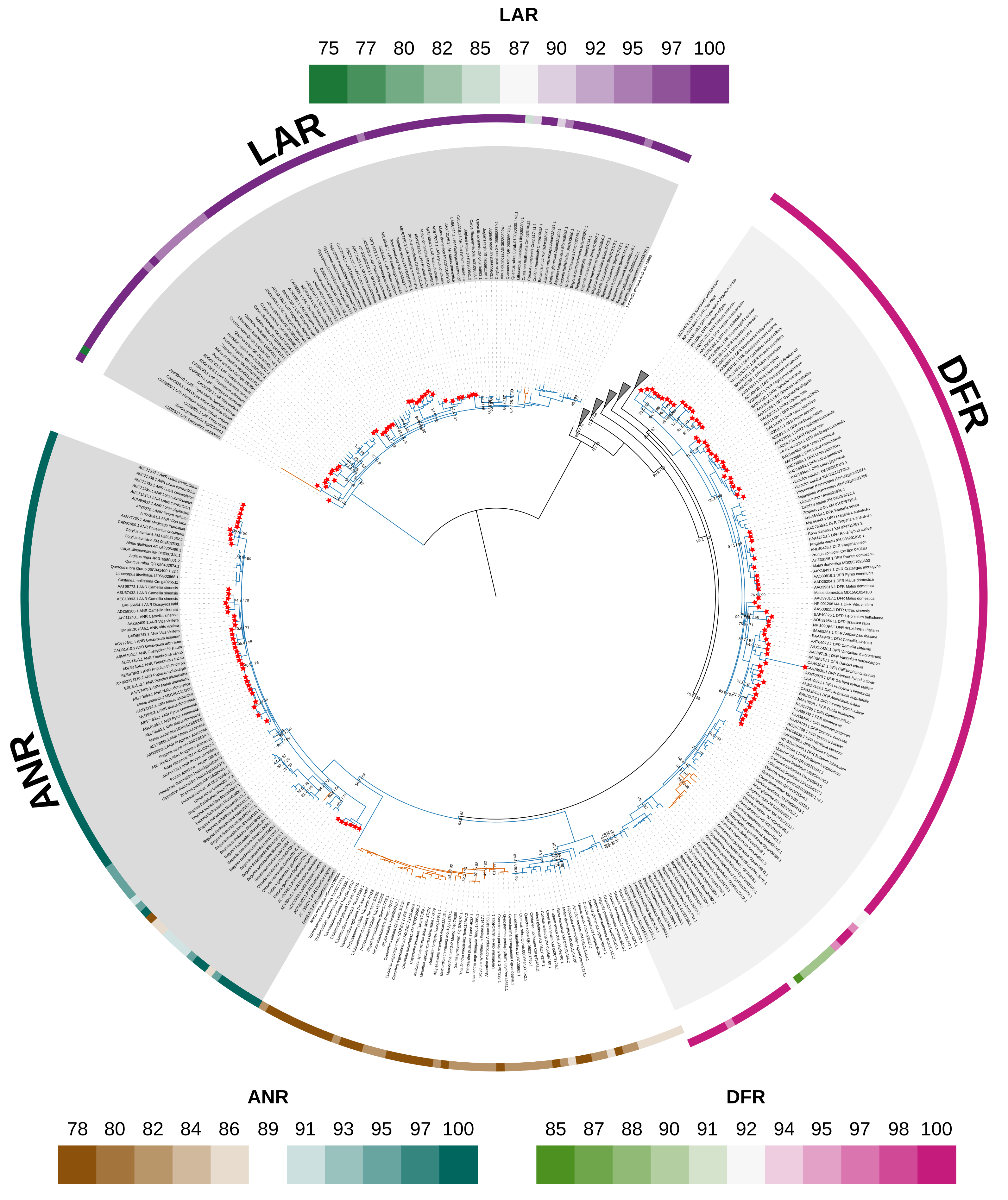

Fig. S8b: Codon-based maximum-likelihood phylogenetic tree of SDR sequences constructed with MUSCLE and IQ-TREE.

The best-fit substitution model selected by Model-Finder was GTR+F+R10. Cucurbitaceae sequences are shown in orange branches, and non-Cucurbitaceae sequences shown in blue. Red stars at the tip of terminal branches indicate functional enzymatic sequences identified in previous studies. Numbers above nodes represent Shimodaira-Hasegawa approximate likelihood ratio test (SH-aLRT) support (%)/ ultrafast bootstrap support (%) based on 1000 replicates. Only percentages below 100 are shown. The outermost colour gradient indicates the percentage of conserved functionally important residues for the corresponding function, thereby reflecting the degree of functional conservation of each sequence. LAR, leucoanthocyanidin reductase; DFR, dihydroflavonol reductase; ANR, anthocyanidin reductase.

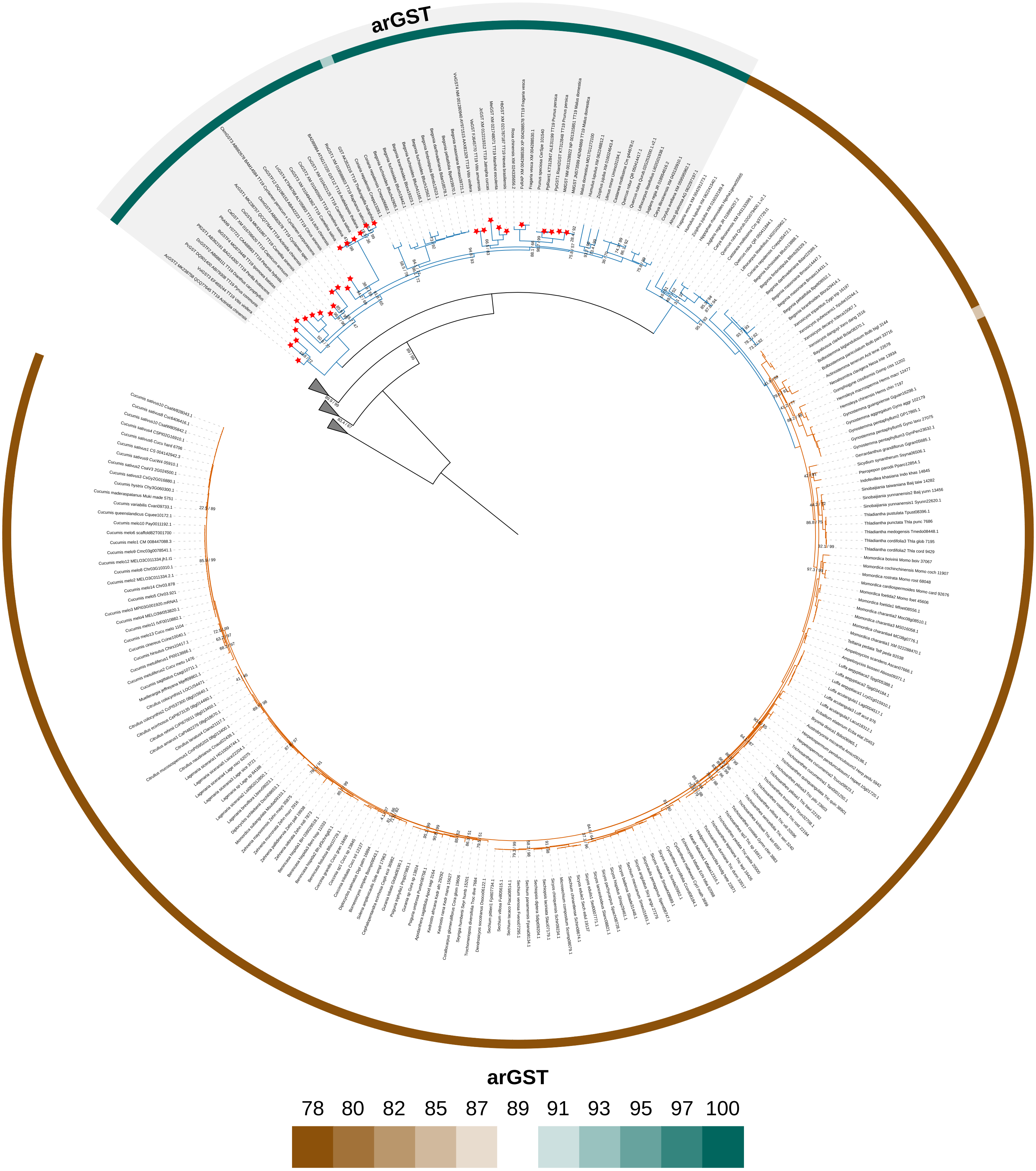

Fig. S9a: Codon-based maximum-likelihood phylogenetic tree of arGST sequences constructed with MAFFT and IQ-TREE.
The best-fit substitution model selected by Model-Finder was TPM3+R6. Cucurbitaceae sequences are shown in orange branches, and non-Cucurbitaceae sequences shown in blue. Red stars at the tip of terminal branches indicate functional enzymatic sequences identified in previous studies. Numbers above nodes represent Shimodaira-Hasegawa approximate likelihood ratio test (SH-aLRT) support (%)/ ultrafast bootstrap support (%) based on 1000 replicates. Only percentages below 100 are shown. The outermost colour gradient indicates the percentage of conserved functionally important residues for the corresponding function, thereby reflecting the degree of functional conservation of each sequence. arGST, anthocyanin-related glutathione S-transferase; TT19, TRANSPARENT TESTA 19

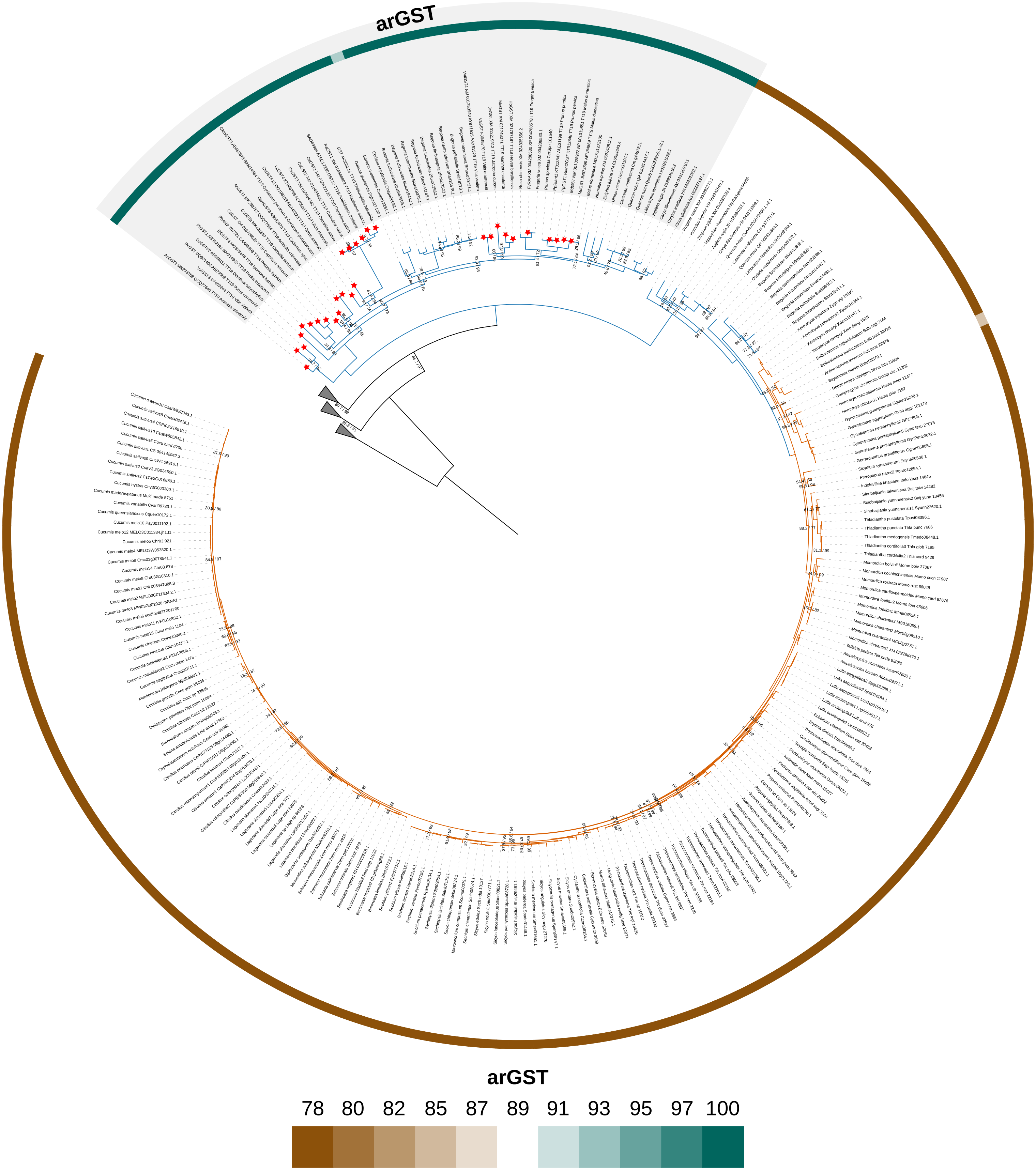

Fig. S9b: Codon-based maximum-likelihood phylogenetic tree of arGST sequences constructed with MUSCLE and IQ-TREE.
The best-fit substitution model selected by Model-Finder was TPM3+I+R6. Cucurbitaceae sequences are shown in orange branches, and non-Cucurbitaceae sequences shown in blue. Red stars at the tip of terminal branches indicate functional enzymatic sequences identified in previous studies. Numbers above nodes represent Shimodaira-Hasegawa approximate likelihood ratio test (SH-aLRT) support (%)/ ultrafast bootstrap support (%) based on 1000 replicates. Only percentages below 100 are shown. The outermost colour gradient indicates the percentage of conserved functionally important residues for the corresponding function, thereby reflecting the degree of functional conservation of each sequence. arGST, anthocyanin-related glutathione S-transferase; TT19, TRANSPARENT TESTA 19

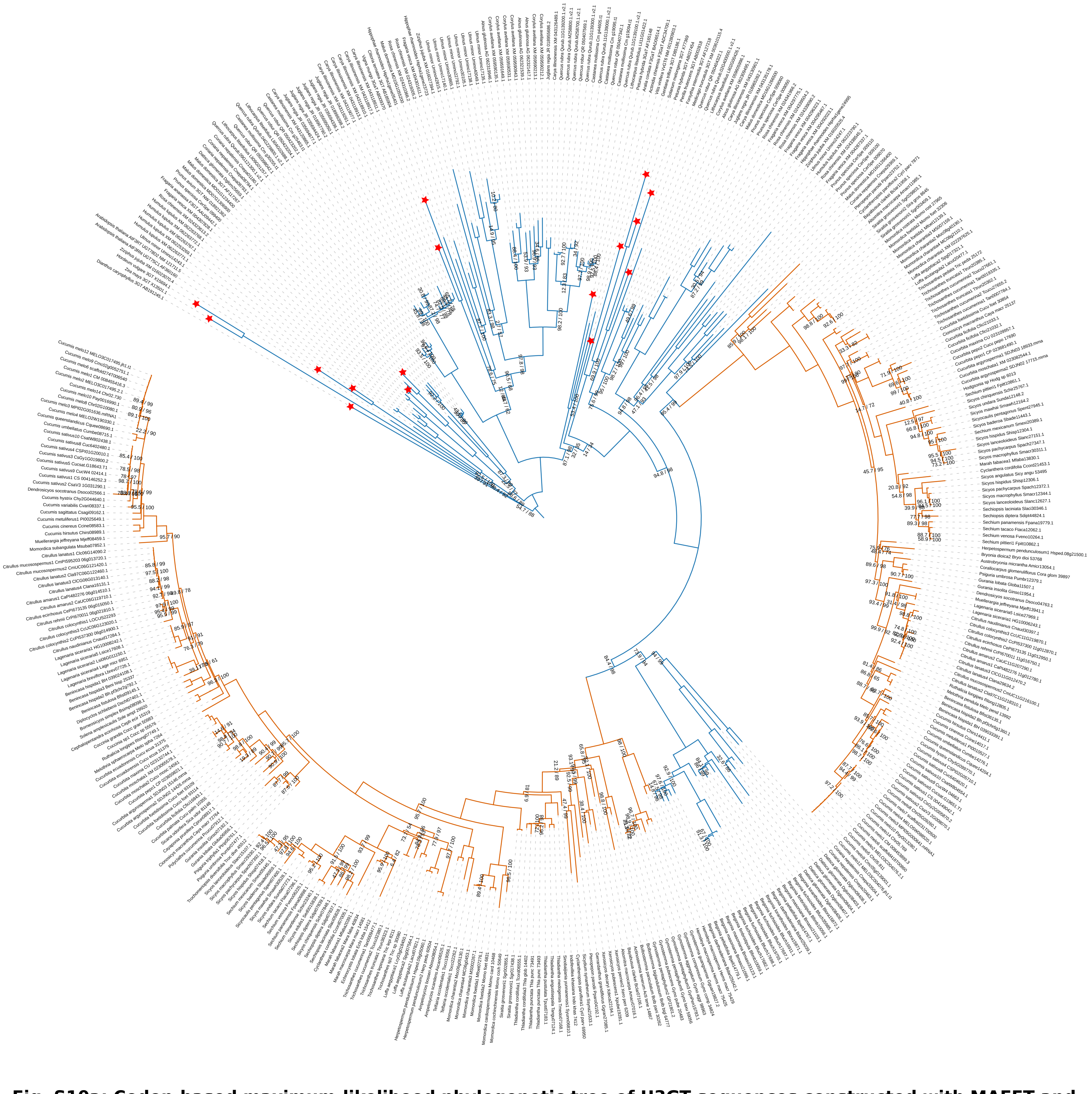

Fig. S10a: Codon-based maximum-likelihood phylogenetic tree of U3GT sequences constructed with MAFFT and IQ-TREE.

The best-fit substitution model selected by Model-Finder was TVMe+I+R6. Cucurbitaceae sequences are shown in orange branches, and non-Cucurbitaceae sequences shown in blue. Red stars at the tip of terminal branches indicate functional enzymatic sequences identified in previous studies. Numbers above nodes represent Shimodaira-Hasegawa approximate likelihood ratio test (SH-aLRT) support (%)/ ultrafast bootstrap support (%) based on 1000 replicates. Only percentages below 100 are shown. U3GT, UDP-glucose-dependent 3-*O*-glucosyltransferase

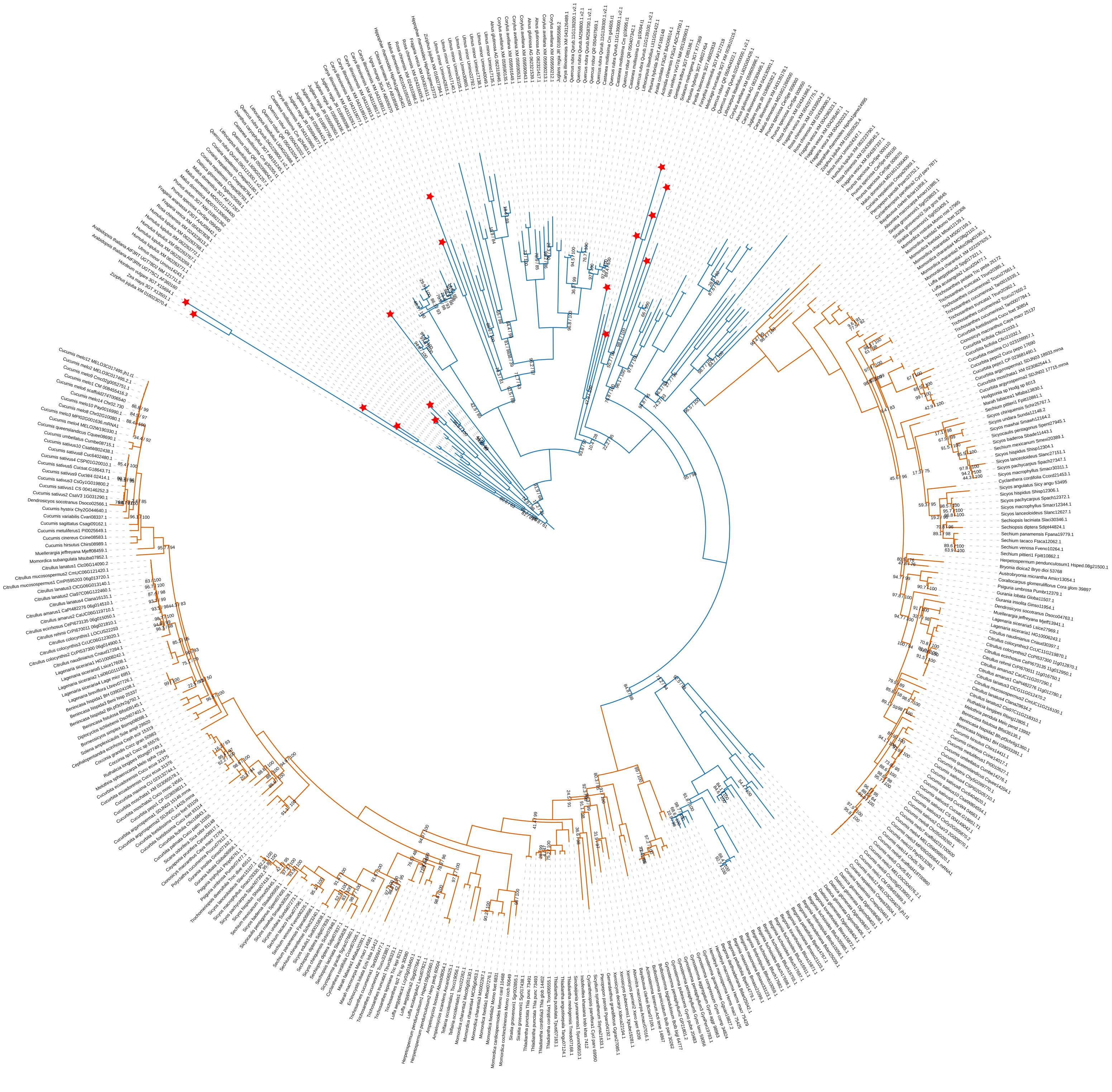

Fig. S10b: Codon-based maximum-likelihood phylogenetic tree of U3GT sequences constructed with MUSCLE and IQ-TREE.

The best-fit substitution model selected by Model-Finder was GTR+F+R6. Cucurbitaceae sequences are shown in orange branches, and non-Cucurbitaceae sequences shown in blue. Red stars at the tip of terminal branches indicate functional enzymatic sequencesidentified in previous studies. Numbers above nodes represent Shimodaira-Hasegawa approximate likelihood ratio test (SH-aLRT) support (%)/ ultrafast bootstrap support (%) based on 1000 replicates. Only percentages below 100 are shown. U3GT, UDP-glucose-dependent 3-*O*-glucosyltransferase

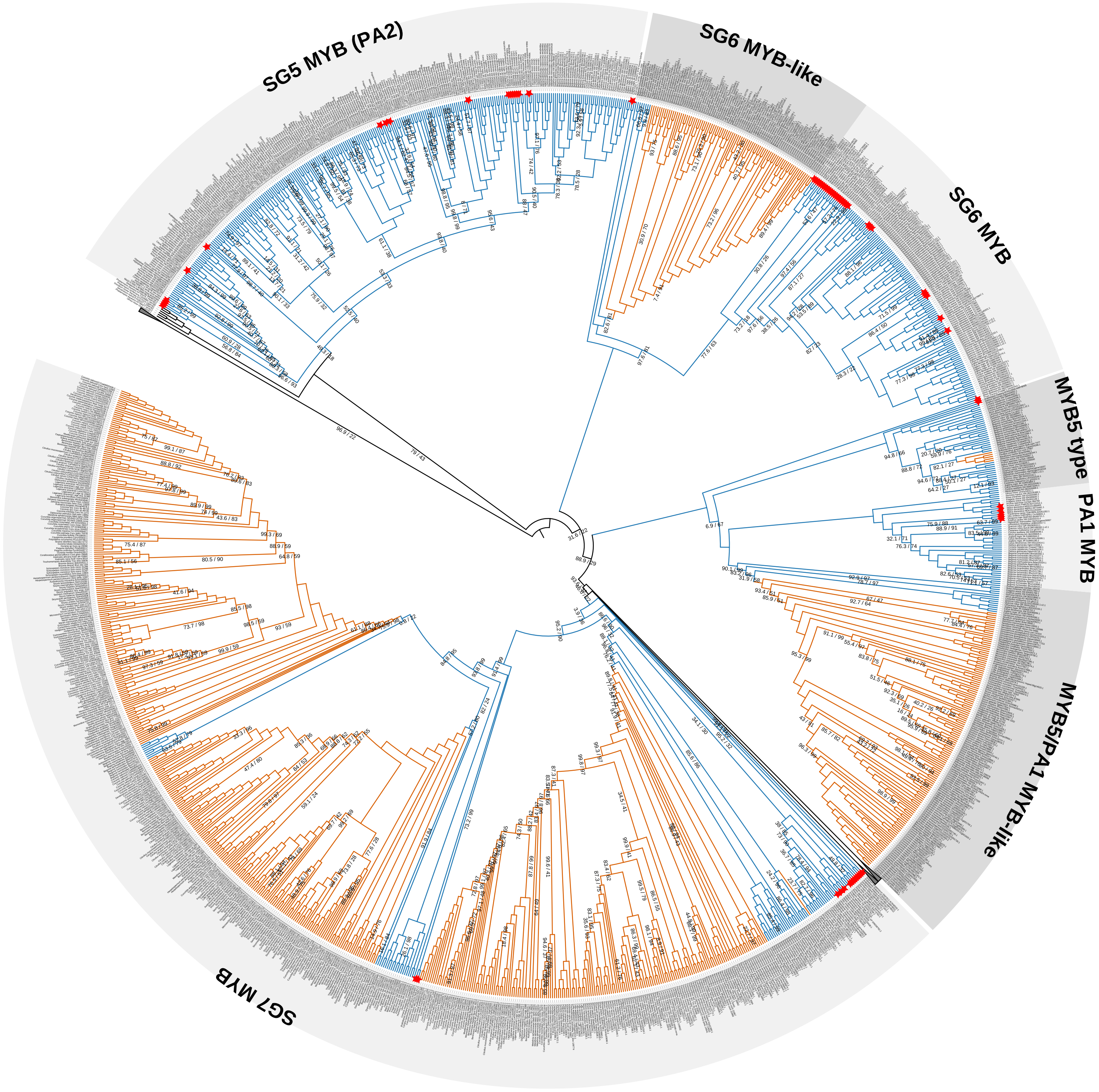

Fig. S11a: Codon-based maximum-likelihood phylogenetic tree of MYB sequences constructed with MAFFT and IQ-TREE.
The best-fit substitution model selected by Model-Finder was GTR+F+ASC+R10. Cucurbitaceae sequences are shown in orange branches, and non-Cucurbitaceae sequences shown in blue. Red stars at the tip of terminal branches indicate functional enzymatic sequences identified in previous studies. Numbers above nodes represent Shimodaira-Hasegawa approximate likelihood ratio test (SH-aLRT)support (%) / ultrafast bootstrap support (%) based on 1000 replicates. Only percentages below 100 are shown. MYB, myeloblastosis; SG, subgroup; PA MYB, proanthocyanidin MYB

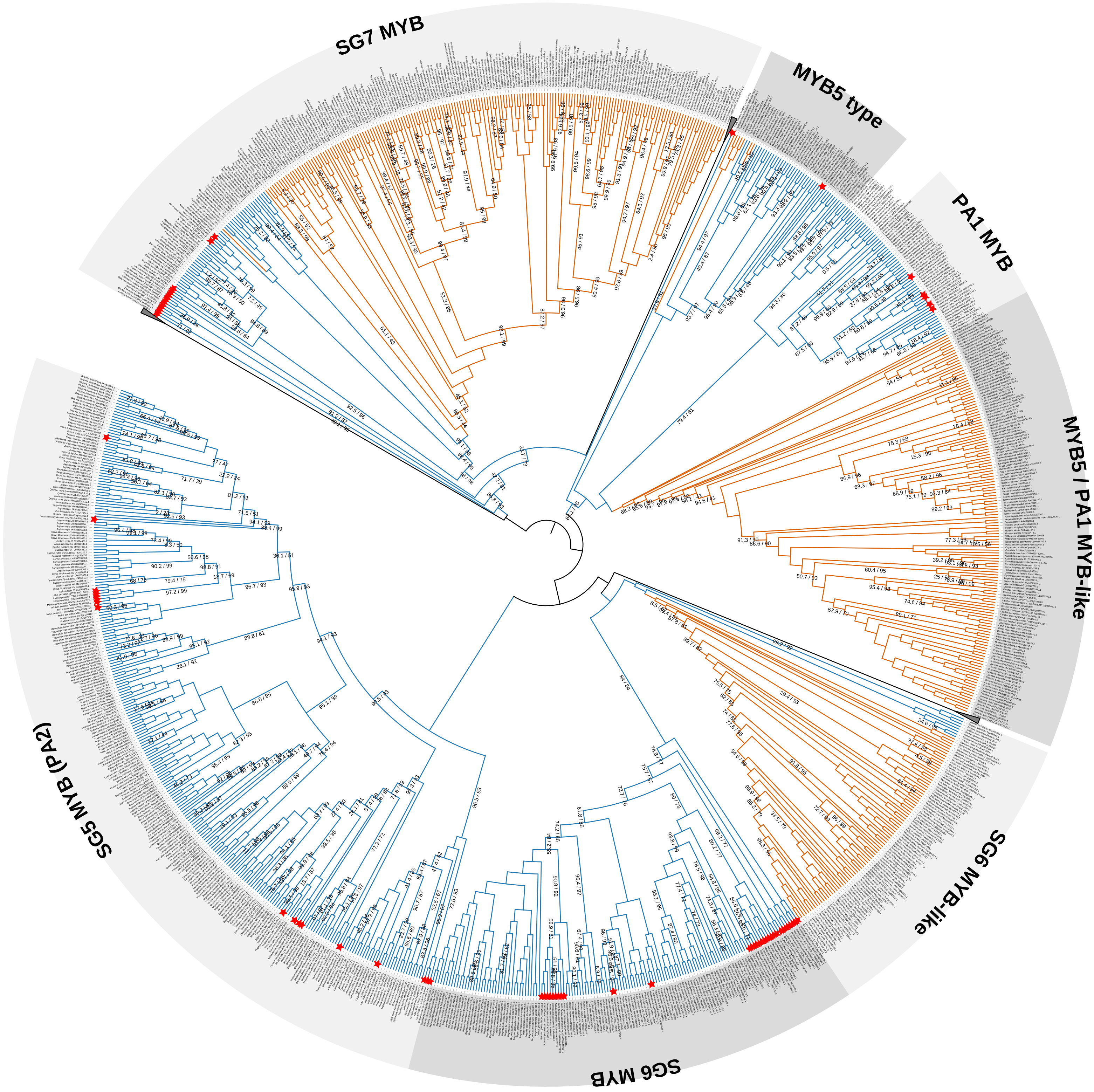

Fig. S11b: Codon-based maximum-likelihood phylogenetic tree of MYB sequences constructed with MUSCLE and IQ-TREE.
The best-fit substitution model selected by Model-Finder was TIM3+F+ASC+R10. Cucurbitaceae sequences are shown in orange branches, and non-Cucurbitaceae sequences shown in blue. Red stars at the tip of terminal branches indicate functional enzymatic sequences identified in previous studies. Numbers above nodes represent Shimodaira-Hasegawa approximate likelihood ratio test (SH-aLRT)support (%) / ultrafast bootstrap support (%) based on 1000 replicates. Only percentages below 100 are shown. MYB, myeloblastosis; SG, subgroup; PA MYB, proanthocyanidin MYB

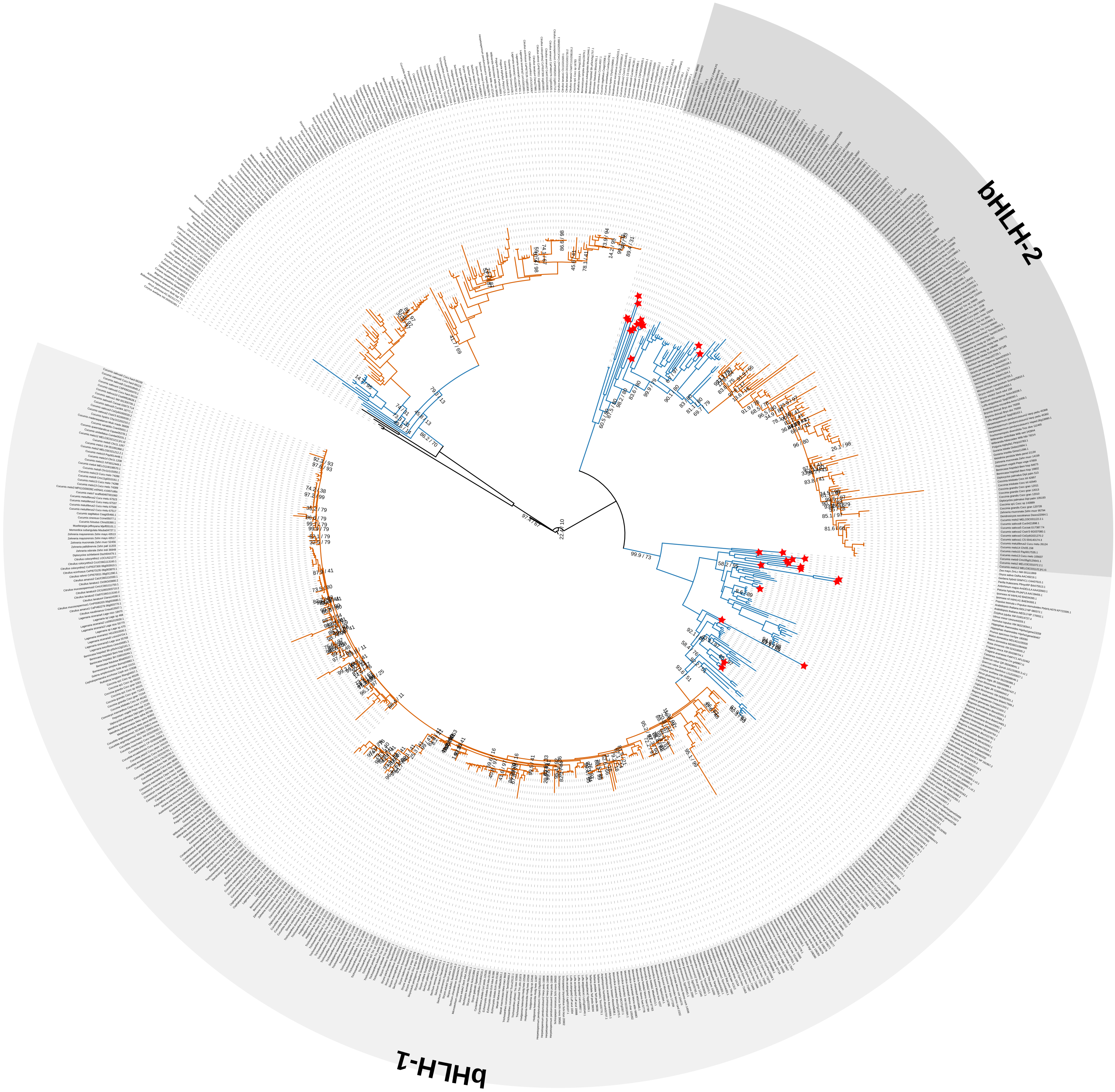

Fig. S12a: Codon-based maximum-likelihood phylogenetic tree of flavonoid-related bHLH sequences constructed with MAFFT and IQ-TREE.

Flavonoid-related bHLHs are regulators of anthocyanin and/or proanthocyanidin pathway and are part of the MBW (MYB-bHLH-WD40) complex. bHLH1 is the *Arabidopsis thaliana* MYC1 ortholog clade while bHLH2 is the *A. thaliana* TT8 ortholog clade. The best-fit substitution model selected by Model-Finder was GTR+F+ASC+R10. Cucurbitaceae sequences are shown in orange branches, and non-Cucurbitaceae sequences shown in blue. Red stars at the tip of terminal branches indicate functional enzymatic sequences identified in previous studies. Numbers above nodes represent Shimodaira-Hasegawa approximate likelihood ratio test (SH-aLRT) support (%) / ultrafast bootstrap support (%) based on 1000 replicates. Only percentages below 100 are shown. bHLH, basic helix-loop-helix.

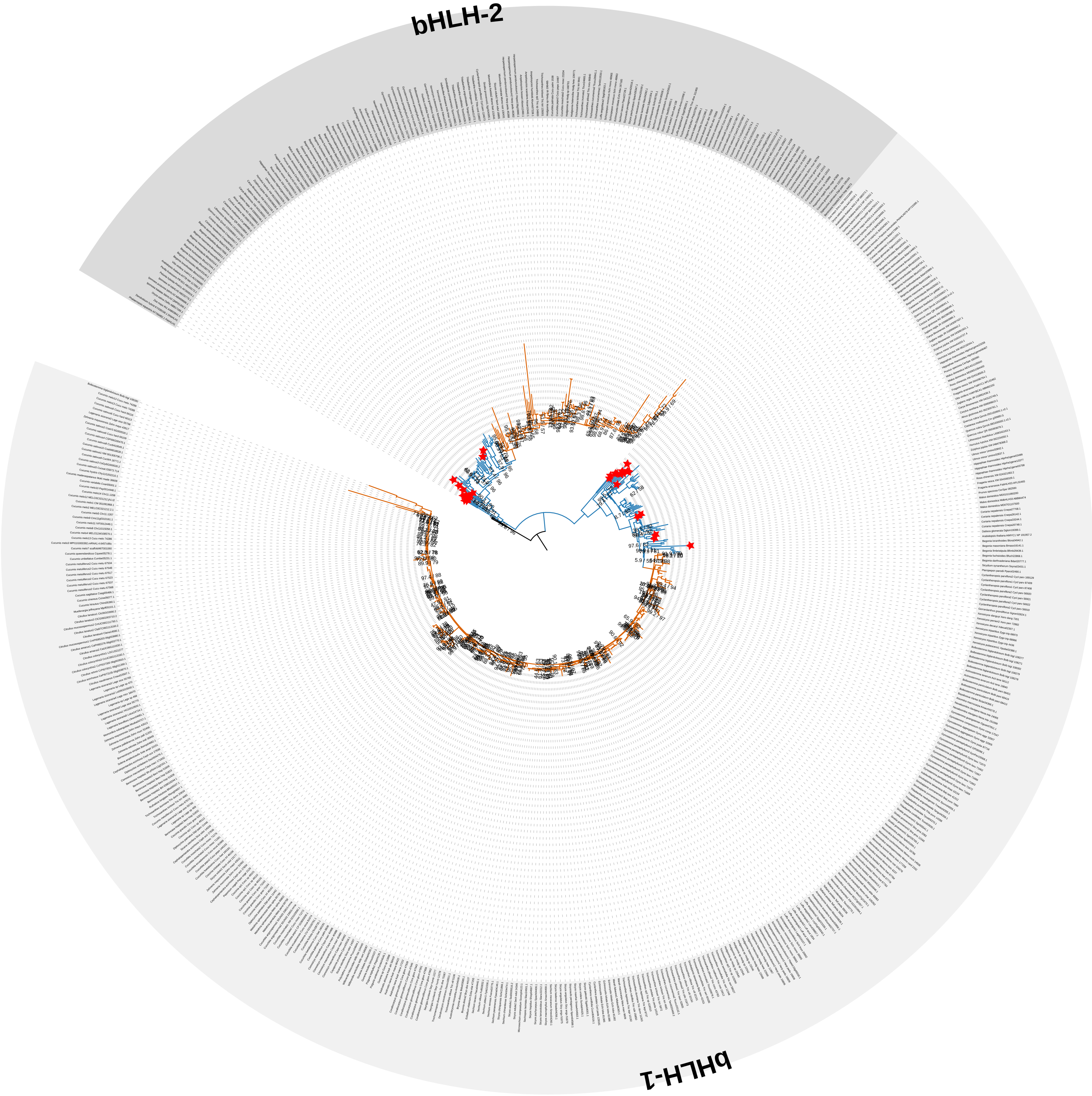

Fig. S12b: Codon-based maximum-likelihood phylogenetic tree of flavonoid-related bHLH sequences constructed with MUSCLE and IQ-TREE.
Flavonoid-related bHLHs are regulators of anthocyanin and/or proanthocyanidin pathway and are part of the MBW (MYB-bHLH-WD40) complex. bHLH1 is the *Arabidopsis thaliana* MYC1 ortholog clade while bHLH2 is the *A. thaliana* TT8 ortholog clade. The best-fit substitution model selected by Model-Finder was GTR+F+ASC+R10. Cucurbitaceae sequences are shown in orange branches, and non-Cucurbitaceae sequences shown in blue. Red stars at the tip of terminal branches indicate functional enzymatic sequences identified in previous studies. Numbers above nodes represent Shimodaira-Hasegawa approximate likelihood ratio test (SH-aLRT) support (%) / ultrafast bootstrap support (%) based on 1000 replicates. Only percentages below 100 are shown. bHLH, basic helix-loop-helix

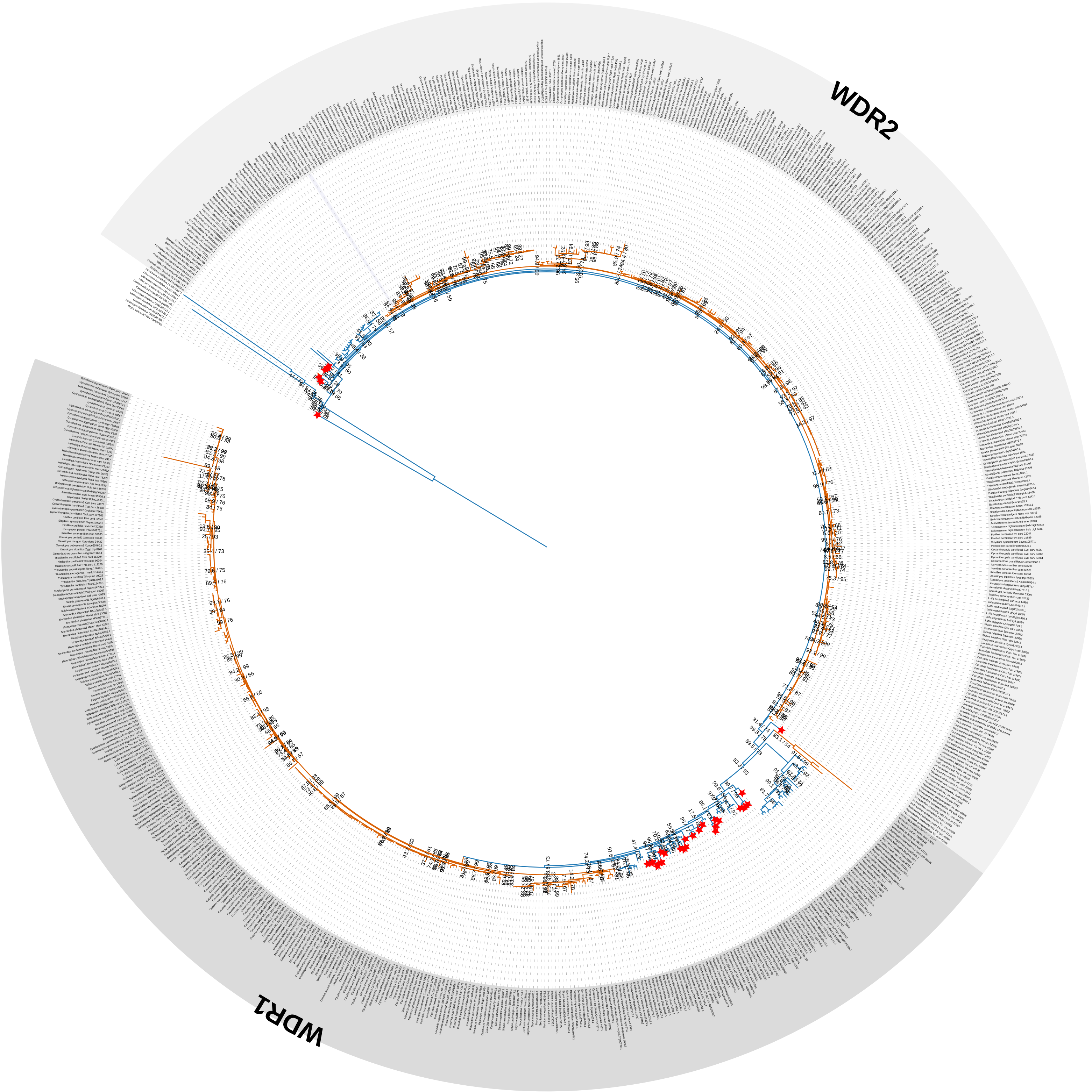

Fig. S13a: Codon-based maximum-likelihood phylogenetic tree of flavonoid-related WD40 sequences constructed with MAFFT and IQ-TREE.

Flavonoid-related WD40s are regulators of anthocyanin and/or proanthocyanidin pathway and are part of the MBW (MYB-bHLH-WD40) complex. WDR1 is the *Arabidopsis thaliana* TTG1 ortholog clade while WDR2 is the *A. thaliana* AN11 ortholog clade. The best-fit substitution model selected by Model-Finder was TIM3+F+R6. Cucurbitaceae sequences are shown in orange branches, and non-Cucurbitaceae sequences shown in blue. Red stars at the start of terminal branch indicate functional enzymatic sequences identified in previous studies. Numbers above nodes represent Shimodaira-Hasegawa approximate likelihood ratio test (SH-aLRT) support (%) / ultrafast bootstrap support (%) based on 1000 replicates. Only percentages below 100 are shown. WDR, WD40-repeat

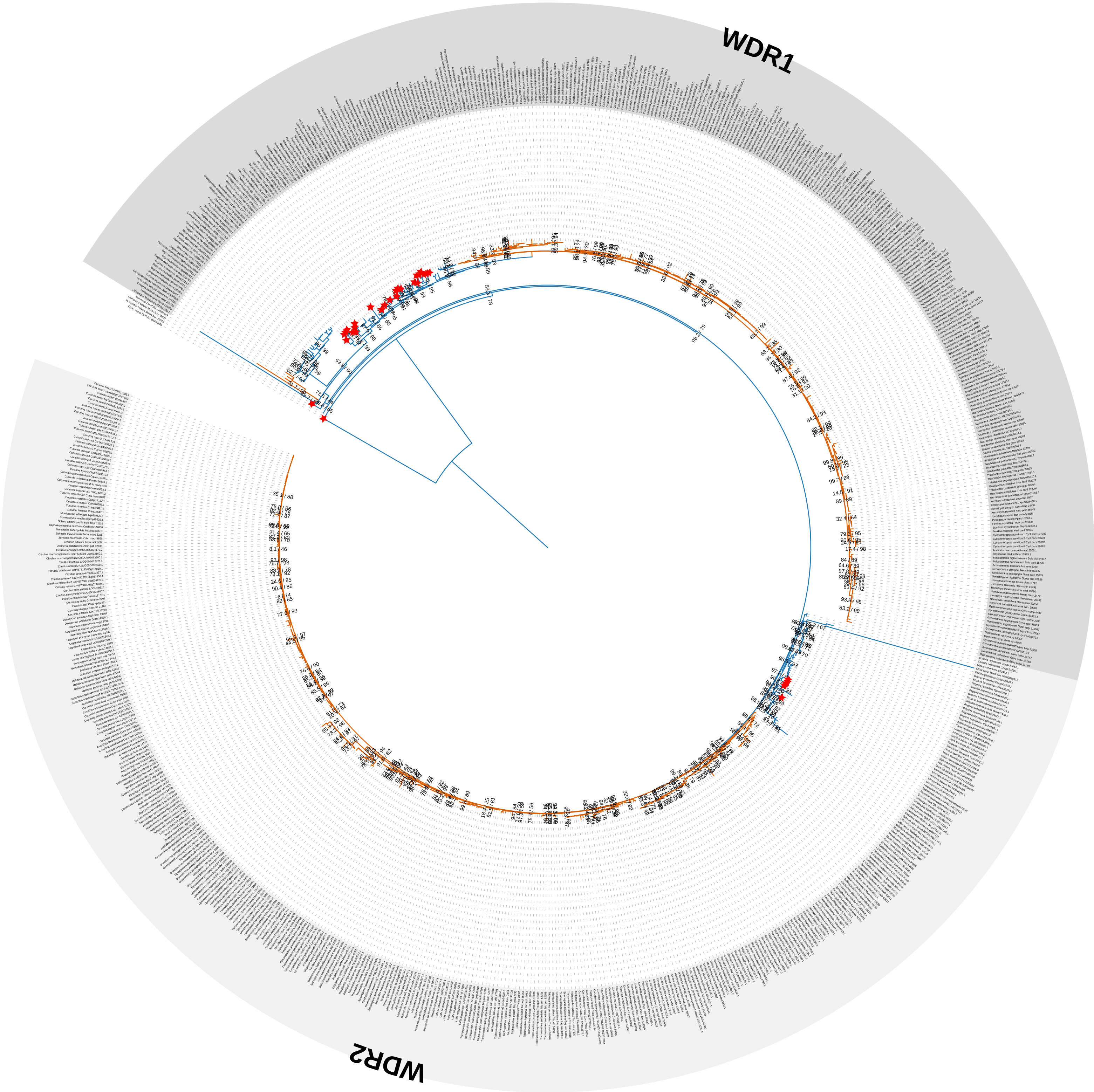

Fig. S13b: Codon-based maximum-likelihood phylogenetic tree of flavonoid-related WD40 sequences constructed with MUSCLE and IQ-TREE.

Flavonoid-related WD40s are regulators of anthocyanin and/or proanthocyanidin pathway and are part of the MBW (MYB-bHLH-WD40) complex. WDR1 is the *Arabidopsis thaliana* TTG1 ortholog clade while WDR2 is the *A. thaliana* AN11 ortholog clade. The best-fit substitution model selected by Model-Finder was TIM3+F+R6. Cucurbitaceae sequences are shown in orange branches, and non-Cucurbitaceae sequences shown in blue. Red stars at the start of terminal branch indicate functional enzymatic sequences identified in previous studies. Numbers above nodes represent Shimodaira-Hasegawa approximate likelihood ratio test (SH-aLRT) support (%) / ultrafast bootstrap support (%) based on 1000 replicates. Only percentages below 100 are shown. WDR, WD40-repeat

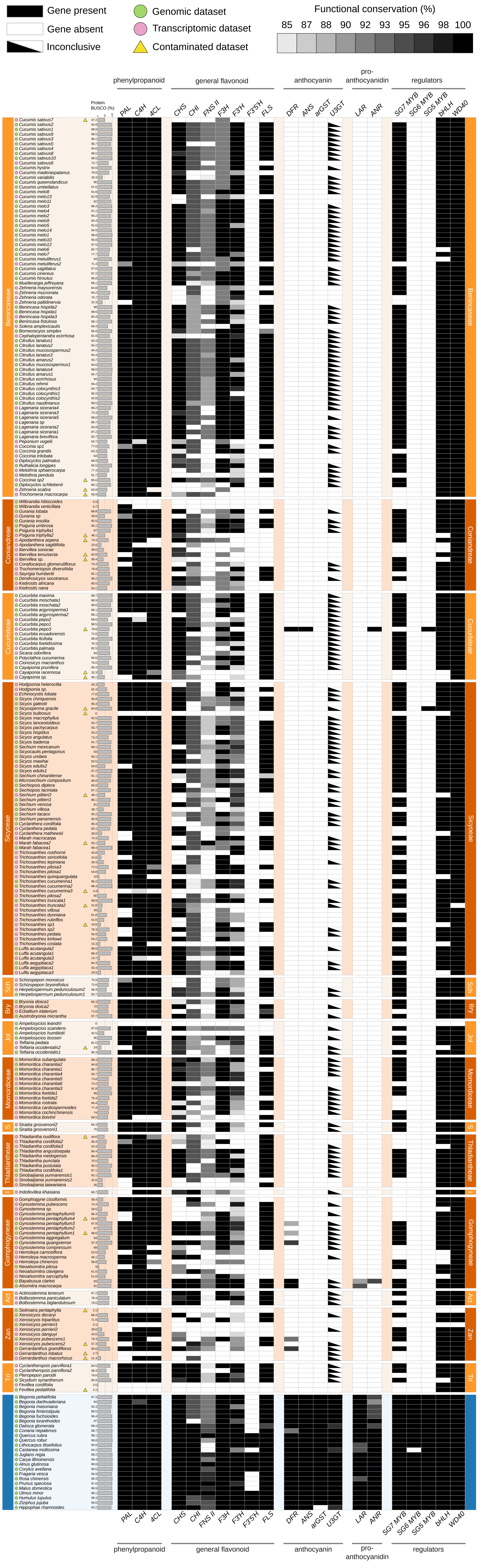

Fig. S14: Presence-absence matrix of flavonoid biosynthesis genes across all datasets analyzed in this study. Outgroup species (blue) and Cucurbitaceae species (orange) are annotated by data source, with genomic datasets shown as green circle and transcriptomics datasets as pink circles. Yellow triangles next to dataset names denote datasets identified as contaminated based on Kraken classification. The percentage of conserved BUSCO genes identified in the polypeptide sequences of each dataset is shown as grey horizontal bars. Gene presence, absence, or putative non-functional sequences are shown as black, white, and grey boxes, respectively. Tribe abbreviations: Tri, Triceratieae; Zan, Zanonieae; Act, Actinostemmateae; I, Indofevilleae; S, Siraitieae; Jol, Joliffieae; Bry, Bryonieae; Sch, Schizopeoneae

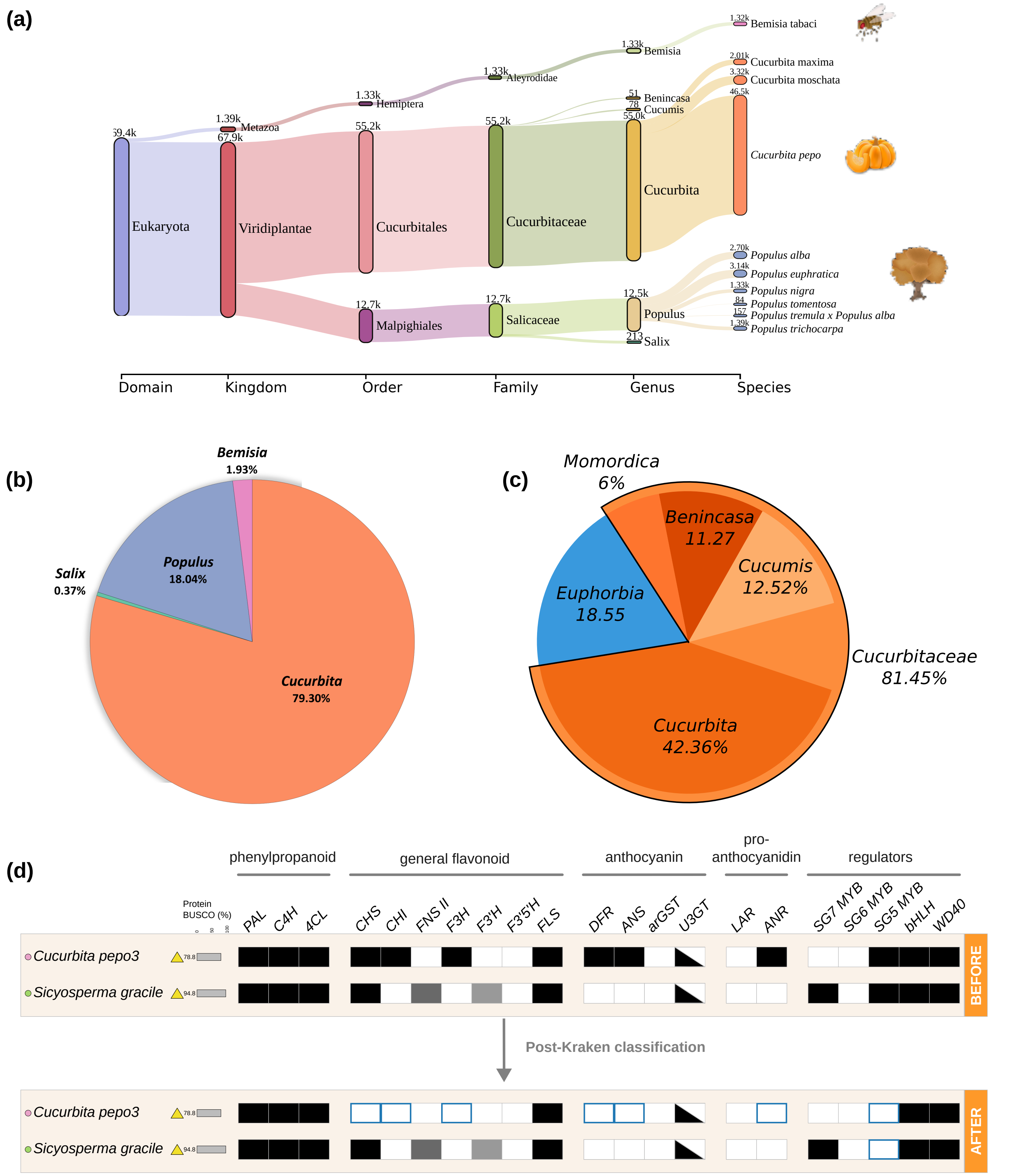

Fig. S15: Contamination profiles of *Cucurbita pepo*3 and *Sicyosperma gracile*

(a) Sankey diagram showing taxonomic classification of *C. pepo*3 sequences from higher taxonomic ranks to species level.
(b) Pie chart summarizing species-level classification of *C. pepo*3 sequences, highlighting contamination from *Populus* species.
(c) Pie chart summarizing species-level classification of *S. gracile* sequences, highlighting contamination from *Euphorbia* species.
(d) Presence-absence matrix of anthocyanin biosynthesis genes in contaminated plant datasets compared with the matrix after contamination check using Kraken. Gene presence is shown as coloured boxes (black/grey) and absence as white boxes. Sequences assigned to non-Cucurbitaceae taxa are removed and highlighted with a blue border, indicating their origin from contaminant species (*Populus* in *C. pepo*3 and *Euphorbia* in *S. gracile*).
These results highlight the importance of contamination screening prior to gene presence-absence analyses, particularly when contamination originates from anthocyanin-pigmented lineages, which can lead to false-positive gene identifications.

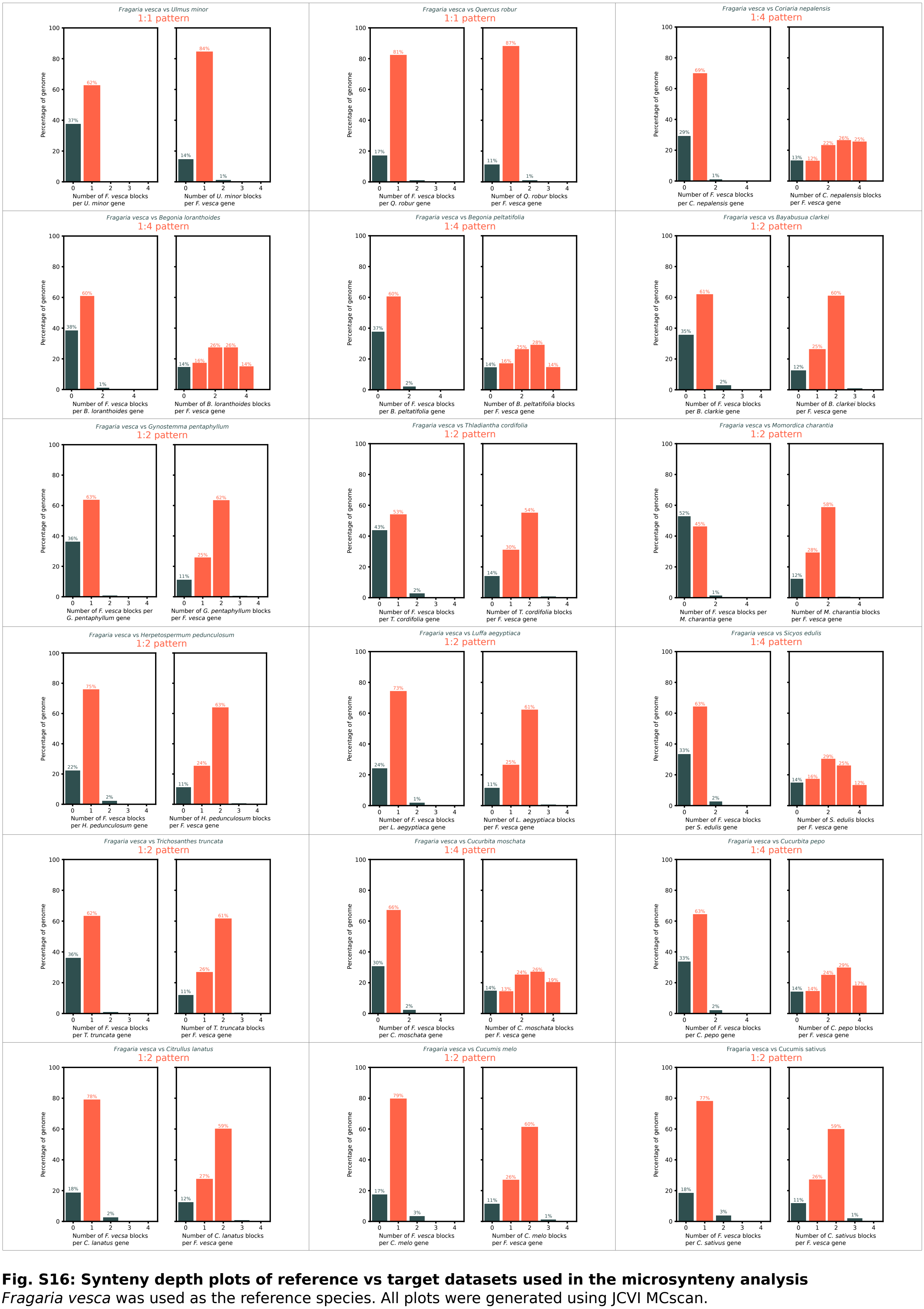

Fig. S16: Synteny depth plots of reference vs target datasets used in the microsynteny analysis
Fragaria vesca was used as the reference species. All plots were generated using JCVI MCscan.

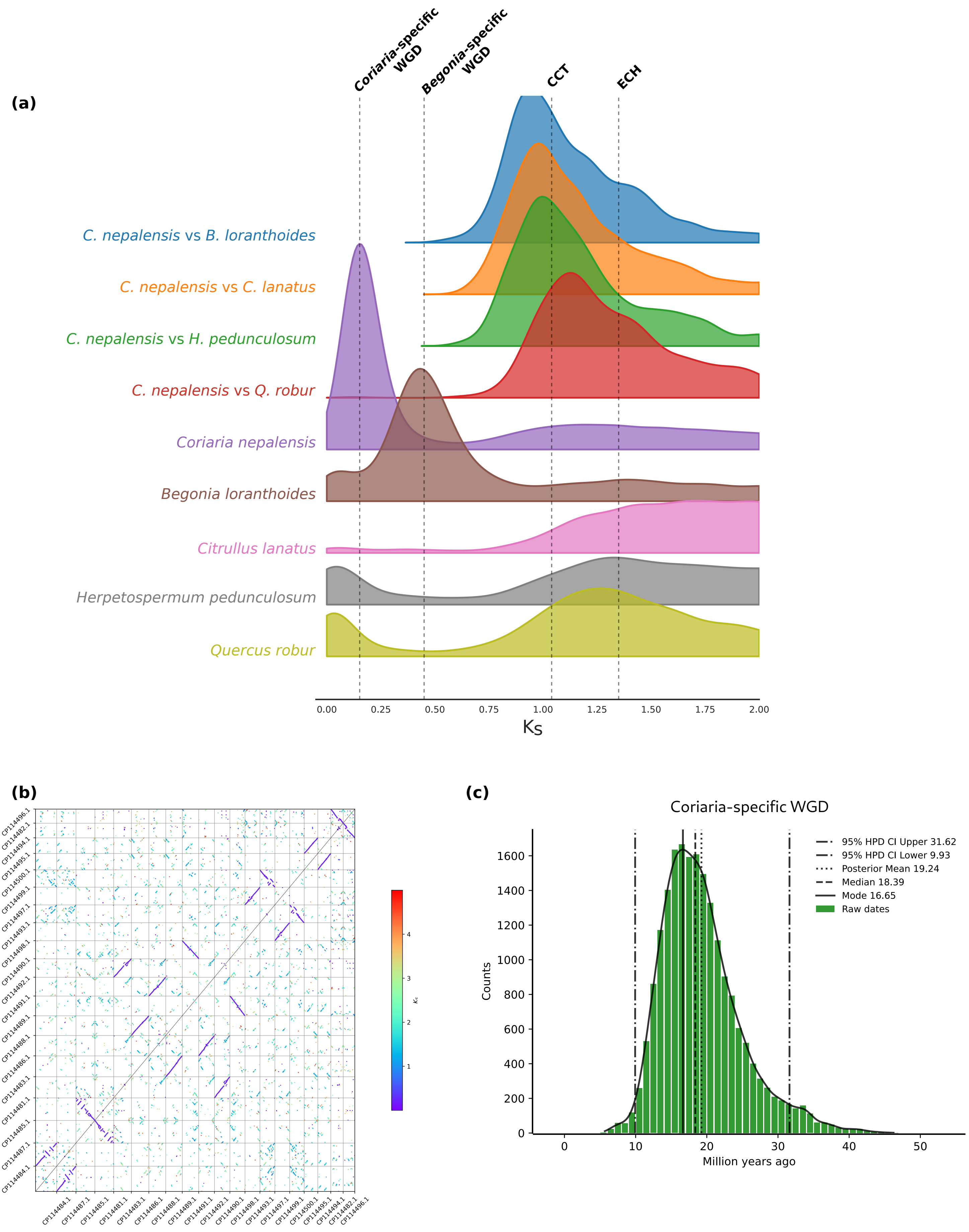

Fig. S17: Whole Genome Duplication (WGD) detection and dating in *Coriaria nepalensis*
(a) The whole paranome *Ks* (synonymous nucleotide substitution rate) distributions of *C. nepalensis* and closely related species, along with evolutionary rate-corrected divergence events (speciation events) based on orthologs bteween these species and *C. nepalensis*.
(b) Intraspecific genome homology dotplots for *C. nepalensis*. Anchor pairs are represented as dots coloured by their associated *Ks* values. Axis show genes on corresponding chromosomes. A large number of homologous genes with a low *Ks* value indicates a recent WGD.
(c) Posterior date distribution, overall mean, and 95% Highest Posterior Density (HPD) of the estimated date of the identified *Coriaria*-specific WGD.
ECH: Core eudicot common hexaploidization; CCT: Cucurbitales common tetraploidization

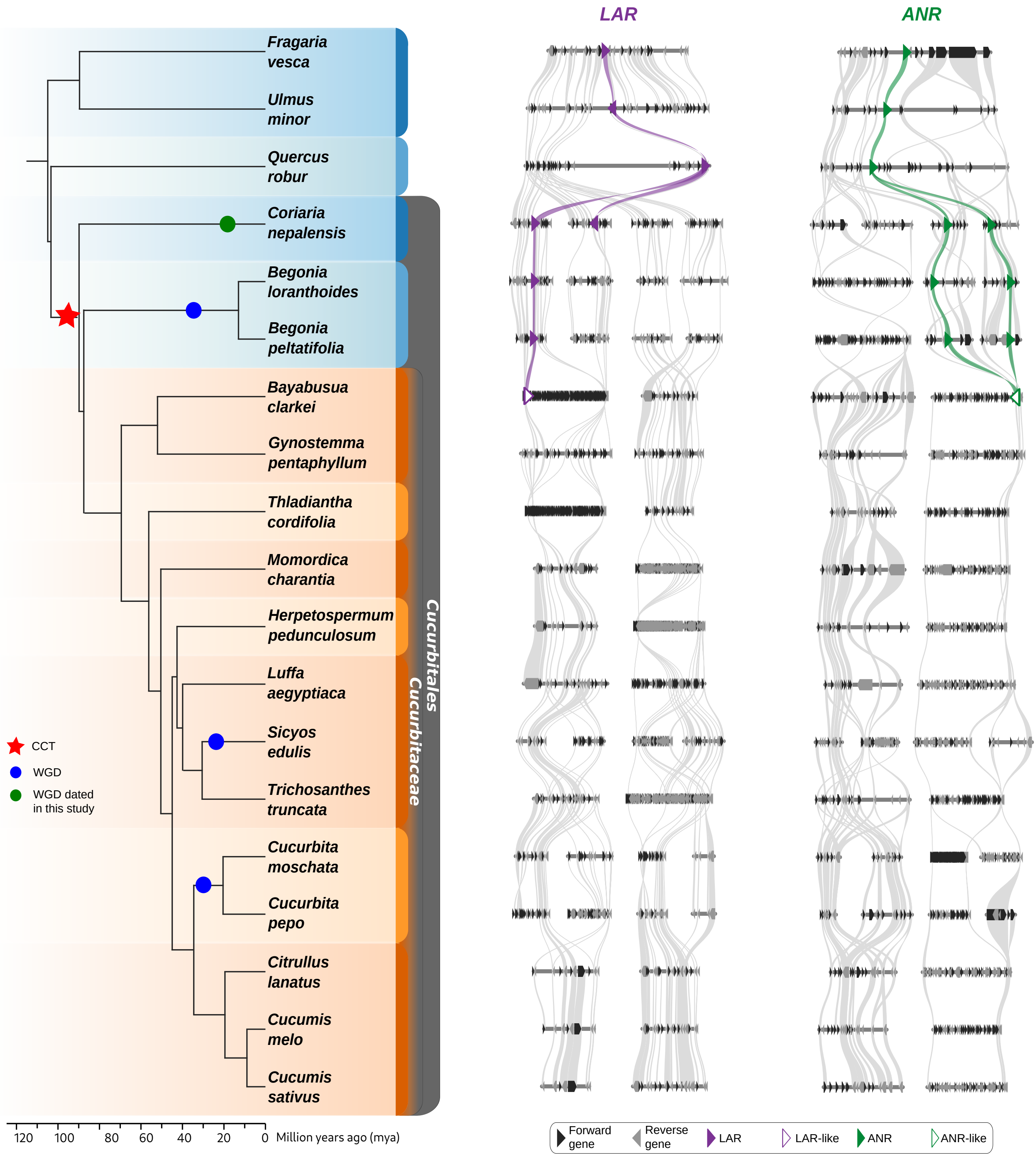

Fig. S18: Microsynteny around LAR and ANR loci.
On the left, the species phylogeny is shown. A red star denotes the Cucurbitales common tetraploidization (CCT) event, while blue circles indicate lineage-specific whole-genome duplications (WGDs): the Begoniaceae common tetraploidization (BCT), the Sicyoeae WGD, and the *Cucurbita*-specific polyploidization (CST). A green circle marks the *Coriaria*-specific WGD dated in this study. On the right, conserved syntenic regions for the proanthocyandin biosynthesis genes, *LAR* (purple) and *ANR* (green) are shown. Genes outlines with unfilled coloured borders represent putatively non-functional copies, based on conserved amino acid residue analysis. Other genes are shown in black (forward strand) and grey (reverse strand). The number of syntenic blocks displayed per species reflects the degree of collinearity relative to *Fragaria vesca* and corresponds to the number of polyploidization events in the evolutionary history of each lineage.

Note: In Begonia species, microsynteny analysis of the ANR locus identified only three syntenic regions corresponding to F. vesca; therefore, only three regions are shown.

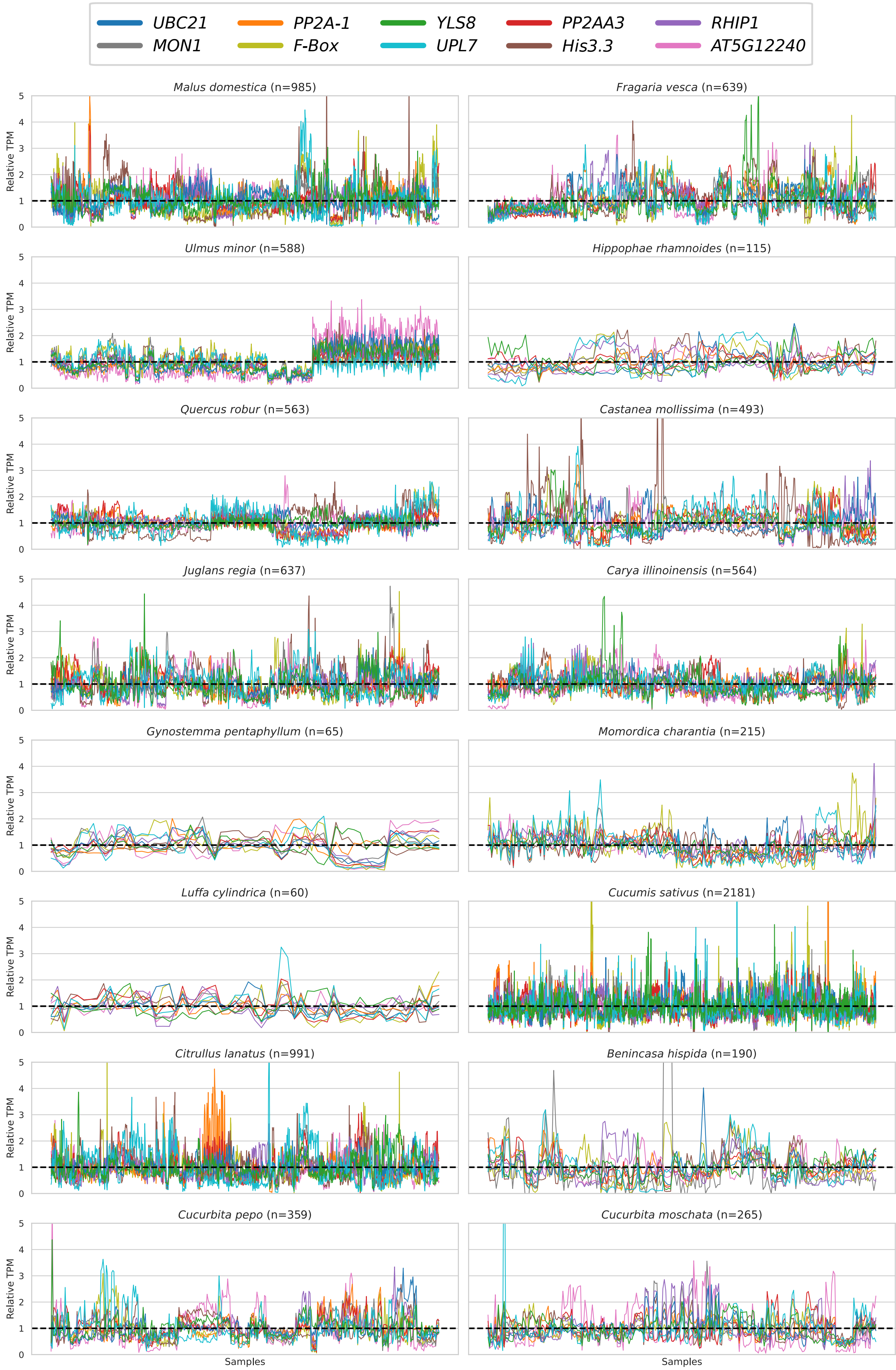

Fig. S19: Relative expression stability of selected reference genes across 16 plant species.
For each species, the relative expression of each reference gene, expressed as transcripts per million (TPM) and calculated as the expression in each sample divided by the mean expression across all samples, is shown across all samples. Sample size (n) for each species is indicated above the plots.

Table S1: Classification of publicly available RNA-seq samples used for comparative expression analyses across Outgroup and Cucurbitaceae species

Numbers indicate the number of RNA-seq samples derived from each tissue type. Samples lacking clear tissue annotations or from mixed tissues in the NCBI metadata are classified as "Unknown".

Outgroups	Leaf	Flower	Fruit	Root	Seed	Stem	Unknown	**Total**
Malus domestica	162	106	495	43	0	108	71	**985**
Fragaria vesca	146	36	192	51	6	39	169	**639**
Ulmus minor	396	0	0	0	0	192	0	**588**
Hippophae rhamnoides	40	0	41	1	10	6	17	**115**
Quercus robur	235	0	0	239	0	6	83	**563**
Castanea mollissima	49	32	12	6	210	0	184	**493**
Juglans regia	198	49	118	17	181	34	40	**637**
Carya illinoinensis	178	38	69	3	101	75	100	**564**
**Outgroup**	**1404**	**261**	**927**	**360**	**508**	**460**	**664**	**4584**

Cucurbitaceae	Leaf	Flower	Fruit	Root	Seed	Stem	Unknown	**Total**
Gynostemma pentaphyllum	28	0	0	9	0	18	10	**65**
Momordica charantia	139	4	16	48	1	1	6	**215**
Luffa aegyptiaca	10	6	31	3	0	6	4	**60**
Cucumis sativus	843	200	351	288	269	120	110	**2181**
Citrullus lanatus	164	45	513	113	22	79	55	**991**
Benincasa hispida	86	0	48	6	42	0	8	**190**
Cucurbita pepo	113	34	77	17	76	38	4	**359**
Cucurbita moschata	52	4	19	69	24	72	25	**265**
**Cucurbitaceae**	**1435**	**293**	**1055**	**553**	**434**	**334**	**222**	**4326**

Table S2: Candidate reference genes evaluated for expression normalization
Reference genes previously reported to show stable expression in *Arabidopsis thaliana* were collected from literature and assessed for expression stability across all species and samples analyzed in this study. Genes were classified as *Selected* based on the stability of their transcript abundance (see Methods). Reasons for exclusion are provided where applicable.

***A. thaliana* ID**	**Gene Name / Function**	**Gene Symbol**	**Status**	**Reason**	**Reference**
AT1G13320	serine/threonine protein phosphatase 2A subunit A3	*PP2AA3*	Selected	Stable transcript abundance across all samples	(Czechowski et al., 2005)
AT1G59830	serine/threonine protein phosphatase PP2A-1 catalytic subunit	*PP2A-1*	Selected	Stable transcript abundance across all samples	(Czechowski et al., 2005)
AT2G28390	monensin sensitivity1; SAND family protein	*MON1*	Selected	Stable transcript abundance across all samples	(Czechowski et al., 2005)
AT3G53090	ubiquitin-protein ligase 7	*UPL7*	Selected	Stable transcript abundance across all samples	(Czechowski et al., 2005)
AT4G26410	RGS1-HXK1 interacting protein 1	*RHIP1*	Selected	Stable transcript abundance across all samples	(Souček *et al.*, 2017)
AT4G40030	histone variant H3	His3.3	Selected	Stable transcript abundance across all samples	(Ferreira et al., 2023)
AT5G08290	yellow-leaf-specific gene 8	*YLS8*	Selected	Stable transcript abundance across all samples	(Czechowski et al., 2005)
AT5G12240	octanoyltransferase	*Expressed -protein*	Selected	Stable transcript abundance across all samples	(Czechowski et al., 2005)
AT5G15710	galactose oxidase/kelch repeat superfamily protein	*Fbox*	Selected	Stable transcript abundance across all samples	(Czechowski et al., 2005)
AT5G25760	ubiquitin-conjugating enzyme 21	*UBC21*	Selected	Stable transcript abundance across all samples	(Czechowski et al., 2005)
AT1G58050	helicase domain-containing protein	*Helicase*	Excluded	Ortholog found; comparatively less stable expression across all samples than the selected ones	(Czechowski et al., 2005)
AT2G32170	methyltransferase gene	*EXPRS*	Excluded	Ortholog found; comparatively less stable expression across all samples than the selected ones	(Czechowski et al., 2005)
AT5G46630	adaptor protein-2 mu-adaptin	*AP2M*	Excluded	Ortholog found; comparatively less stable expression across all samples than the selected ones	(Czechowski et al., 2005)
AT4G27960	ubiquitin-conjugating enzyme 9	*UBC9*	Excluded	Ortholog found; comparatively less stable expression across all samples than the selected ones	(Czechowski et al., 2005)
AT4G33380	adenosine tRNA methylthiotransferase	*Expressed -protein*	Excluded	Ortholog found; comparatively less stable expression across all samples than the selected ones	(Czechowski et al., 2005)
AT4G34270	TAP42 interacting protein of 41 kda	*TIP41*	Excluded	Ortholog found; comparatively less stable expression across all samples than the selected ones	(Czechowski et al., 2005)
AT1G50010	tubulin alpha-2 chain	*TUA2*	Excluded	Ortholog not identified in *C. illinoinensis* and *J. regia*	(Ferreira et al., 2023)
AT3G02470	S-adenosylmethionine decarboxylase	*SAMDC*	Excluded	Ortholog not identified in *L. aegyptiaca*	(Ferreira et al., 2023)
AT3G18780	actin 2	*ACT2*	Excluded	Ortholog not identified in *F. vesca*	(Souček *et al.*, 2017)
AT4G05320	ubiquitin 10	*UBQ10*	Excluded	Ortholog not identified in *F. vesca*, *H. rhamnoides*, and *Q. robur*	(Souček *et al.*, 2017)
AT1G62930	RNA processing factor 3	*RPF3*	Excluded	Ortholog not identified in *L. aegyptiaca*, *M. charantia*, *C. sativus*, *B. hispida*, *C. lanatus*, *C. pepo*, *C. moschata*	(Czechowski et al., 2005)
AT4G38070	transcription factor bHLH131-like protein	*bHLH131*	Excluded	Ortholog not identified in *U. minor* and *G. pentaphyllum*	(Czechowski et al., 2005)
AT5G09810	actin 7	*ACT7*	Excluded	Ortholog not identified in *H. rhamnoides*	(Ferreira et al., 2023)
AT1G30950	unusual floral organs	*UFO*	Excluded	Ortholog not identified in *L. aegyptiaca*	(Hong *et al.*, 2010)
AT5G55840	PPR superfamily protein	*PPR*	Excluded	Ortholog not identified in *U. minor* and *H. rhamnoides*	(Czechowski et al., 2005)
AT5G60390	elongation factor 1-alpha 4	*EF-1α*	Excluded	Failed to identify clear ortholog clade	(Souček *et al.*, 2017)
AT5G62690	tubulin beta chain 2	*TUB2*	Excluded	Failed to identify clear ortholog clade	(Hong *et al.*, 2010)
AT1G07920	elongation factor 1-alpha 3	*EF-1α3*	Excluded	Failed to identify clear ortholog clade	(Souček *et al.*, 2017)
AT1G13440	glyceraldehyde-3-phosphare dehydrogenase	*GAPDH*	Excluded	Failed to identify clear ortholog clade	(Czechowski et al., 2005)
AT1G14320	suppressor of ACAULIS 52	*SAC52*	Excluded	Failed to identify clear ortholog clade	(Ferreira et al., 2023)
AT3G01150	polypyrimidine tract-binding protein	*PTB*	Excluded	Failed to identify clear ortholog clade	(Czechowski et al., 2005)
AT4G36800	RUB1 conjugating enzyme 1	*RCE1*	Excluded	Failed to identify clear ortholog clade	(Ferreira et al., 2023)

Table S3: Reference gene orthologs used for expression normalisation across species

For each of the ten selected reference genes, corresponding orthologous gene models used for expression analysis are listed for each species.

	PP2AA3	*PP2A-1*	*MON1*	*UPL7*	*RHIP1*	*His3.3*	*YLS8*	*Expressed-protein*	*Fbox*	*UBC21*
Arabidopsis thaliana	**AT1G13320**	**AT1G59830**	**AT2G28390**	**AT3G53090**	**AT4G26410**	**AT4G40030**	**AT5G08290**	**AT5G12240**	**AT5G15710**	**AT5G25760**
Malus domestica	MD13G1044500, MD16G1045400, MD06G1214700, MD14G1225600	MD13G1083600, MD16G1083000, MD14G1189900, MD06G1183800	MD03G1118200, MD11G1136600	MD12G1101300, MD14G1095600	MD11G1001100	MD15G1320600, MD15G1373700	MD16G1003600, MD13G1003900	MD11G1174500	MD09G1076600	MD08G1116800, MD15G1096300
Fragaria vesca	XM_004300486.1, XM_004297079.1	XM_004299571.1, XM_004297640.1	XM_004293004.1	XM_004303006.1	XM_004294885.1	XM_004299624.1, XM_004291779.1, XM_004288352.1, XM_004288116.1, XM_004287165.1	XM_004293850.1, XM_004297683.1	XM_004291484.1	XM_004304138.1	XM_004291147.1
Ulmus minor	Umino36473.1, Umino46550.1	Umino45791.1, Umino35680.1	Umino28814.1	Umino50448.1	Umino18891.1	Umino39484.1, Umino05842.1	Umino51084.1	Umino02358.1	Umino04777.1	Umino06817.1
Hippophae rhamnoides	Hiprha1gene00320	Hiprha1gene16857, Hiprha1gene18525, Hiprha1gene25511, Hiprha1gene30501	Hiprha1gene24930, Hiprha1gene29846	Hiprha1gene27518	Hiprha1gene16323	Hiprha1gene30621, Hiprha1gene29044, Hiprha1gene27419, Hiprha1gene04942, Hiprha1gene04589	Hiprha1gene18545	Hiprha1gene27925	Hiprha1gene21641, Hiprha1gene20154	Hiprha1gene15154, Hiprha1gene14277
Quercus robur	QR_050385359.1, QR_050402249.1	QR_050400796.1, QR_050420939.1	QR_050430911.1, QR_050388804.1	QR_050387415.1	QR_050421740.1	QR_050436377.1, QR_050435275.1, QR_050411659.1	QR_050383967.1	QR_050427206.1	QR_050414474.1	QR_050409355.1
Castanea mollisima	Cm_g15912.t1	Cm_g6662.t1, Cm_g5221.t1	Cm_g21920.t1	Cm_g49605.t1	Cm_g49767.t1	Cm_g11307.t1	Cm_g20732.t1	Cm_g4925.t1	Cm_g17260.t1	Cm_g2572.t1
Juglans regia	JR_035685846.1, JR_018953794.2, JR_018974404.2	JR_018963562.2, JR_035684549.1	JR_018963979.2, JR_035690214.1	JR_018988558.2	JR_018981994.2	JR_018993457.2, JR_018992219.2, JR_018978364.2, JR_018965606.2	JR_018974444.2, JR_018961779.2	JR_035690453.1	JR_018961389.2	JR_035693748.1
Carya illinoinensis	XM_043109563.1, XM_043105771.1	XM_043128385.1, XM_043124679.1	XM_043099773.1, XM_043103013.1	XM_043086155.1	XM_043101211.1	XM_043133397.1, XM_043120035.1, XM_043115527.1, XM_043104386.1	XM_043122040.1, XM_043094639.1	XM_043103934.1	XM_043114513.1	XM_043097133.1
Gynostemma pentaphyllum2	GP26543.1, GP39264.1, GP39191.1	GP17806.1, GP24603.1, GP24730.1	GP03979.1, GP03839.1	GP08413.1	GP22475.1, GP22360.1	GP18640.1, GP36564.1, GP36565.1	GP26132.1	GP35933.1, GP36792.1	GP02000.1	GP18502.1
Momordica charantia2	Moc09g37880.1	Moc09g39880.1, Moc08g32520.1	Moc03g22040.1	Moc05g30760.1	Moc06g09890.1	Moc08g02280.1	Moc08g39760.1, Moc09g34770.1	Moc02g01960.1	Moc04g37150.1	Moc08g03380.1
Luffa aegyptiaca1	Lcy13g004410.1	Lcy01g020090.1, Lcy13g002280.1	Lcy10g004280.1	Lcy06g020730.1	Lcy03g010700.1	Lcy11g009740.1	Lcy13g008340.1	Lcy07g017940.1	Lcy09g003070.1	Lcy01g011310.1
Cucumis sativus1	CS_011657465.2	CS_004135164.3, CS_004146712.3	CS_004139400.3	CS_031882877.1	CS_004144016.3	CS_031889847.1, CS_031889801.1, CS_031889800.1, CS_004152577.3, CS_004146827.3	CS_031881493.1, CS_004143570.3	CS_004140136.3	CS_031885920.1	CS_004150235.3
Citrullus lanatus2	Cla97C05G104770.1	Cla97C05G106960.1, Cla97C08G151900.1	Cla97C01G012740.1	Cla97C10G202640.2	Cla97C03G064890.2	Cla97C08G154270.1	Cla97C02G038590.1, Cla97C02G039800.1	Cla97C02G048780.1	Cla97C11G211810.2	Cla97C08G155510.2
Benincasa hispida1	BH_039036531.1	BH_039035994.1, BH_039028886.1	BH_039038527.1	BH_039048935.1	BH_039024044.1	BH_039030074.1	BH_039046947.1	BH_039044486.1	BH_039034704.1	BH_039030857.1
Cucurbita pepo1	CP_023680913.1, CP_023686598.1	CP_023658059.1, CP_023690273.1, CP_023681854.1	CP_023664294.1	CP_023683460.1	CP_023661056.1	CP_023675962.1, CP_023691263.1, CP_023697253.1	CP_023657340.1, CP_023676953.1, CP_023687468.1	CP_023676616.1	CP_023657641.1, CP_023693801.1	CP_023680983.1, CP_023689814.1
Cucurbita moschata1	XM_023101179.1, XM_023089599.1	XM_023095857.1, XM_023090726.1, XM_023101151.1	XM_023109205.1	XM_023091022.1	XM_023105465.1	XM_023073716.1, XM_023086482.1, XM_023106719.1	XM_023077391.1, XM_023101318.1, XM_023086844.1, XM_023072211.1	XM_023102091.1	XM_023084181.1, XM_023109268.1	XM_023095212.1, XM_023106552.1

Table S4: Genes used for expression analyses of carotenoid biosynthesis genes in outgroups and Cucurbitaceae

For each species, gene IDs corresponding to carotenoid biosynthesis enzymes are listed. Functional conservation (FC, %) indicates the percentage of conserved amino acid residues required for the annotated enzyme function.

*For CrtISO, expression values of *CrtISO1* and *CrtISO2* gene candidates were added to derive total CrtISO expression shown in Fig. 5.

	Z-ISO		ZDS		CrtISO1*		CrtISO2*		LCYE	
	Gene ID	FC (%)	Gene ID	FC (%)	Gene ID	FC (%)	Gene ID	FC (%)	Gene ID	FC (%)
Malus domestica	MD04G1028100	100	MD04G1220900	100	MD14G1044400	100	MD08G1243700	100	MD02G1083500	100
			MD12G1237300	100						
Fragaria vesca	XM_004297980.1	100	XM_004301977.1	100	XM_004303334.1	100	XM_004301509.1	100	XM_004287534.1	100
Ulmus minor	Umino51417.1	100	Umino29712.1	100	Umino49671.1	100	Umino30776.1	100	Umino33628.1	100
			Umino29693.1	100						
Hippophae rhamnoides	Hiprha1gene09348	100	Hiprha1gene16000	100	Hiprha1gene17015	100	Hiprha1gene16344	100	Hiprha1gene10883	100
									Hiprha1gene03401	100
Quercus robur	QR_050399978.1	100	QR_050419023.1	100	QR_050388536.1	100	QR_050416925.1	100	QR_050414224.1	100
							QR_050416927.1	100	QR_050414223.1	100
Castanea mollissima	Cm_g24958.t1	100	Cm_g17152.t1	100	Cm_g36140.t1	100	Cm_g28965.t1	100	Cm_g21153.t1	100
Juglans regia	JR_018972132.2	100	JR_018990301.2	100	JR_018993267.2	100	JR_018955462.2	100	JR_018990653.2	100
							JR_018955463.2	100		
Carya illinoinensis	XM_043115075.1	100	XM_043133515.1	100	XM_043084812.1	100	XM_043127913.1	100	XM_043121056.1	100
***Gynostemma pentaphyllum*2**	GP18982.1	100	GP23721.1	100	GP08653.1	100	GP36474.1	100	GP11616.1	100
			GP23785.1	100						
***Momordica charantia*2**	Moc05g08940.1	100	Moc06g26840.1	100	Moc05g29080.1	100	Moc10g05510.1	100	Moc08g45730.1	100
***Luffa aegyptiaca*1**	Lcy10g009130.1	100	Lcy03g004530.1	100	Lcy06g018940.1	91.6	Lcy11g011320.1	100	Lcy08g018900.1	100
***Cucumis sativus*1**	CS_004152053.3	100	CS_004142474.3	100	CS_004136487.3	100	CS_011652269.2	100	CS_004141124.3	100
***Citrullus lanatus*2**	Cla97C07G142740.2	100	Cla97C06G118930.2	100	Cla97C10G200950.2	100	Cla97C10G190930.2	100	Cla97C11G208040.2	100
***Benincasa hispida*1**	BH_039026618.1	100	BH_039023482.1	100	BH_039047825.1	100	BH_039049488.1	100	BH_039035238.1	100
***Cucurbita pepo*1**	CP_023684933.1	100	CP_023674191.1	100	CP_023683621.1	100	CP_023697681.1	100	CP_023682807.1	100
									CP_023691131.1	100
***Cucurbita moschata*1**	XM_023076947.1	100	XM_023077721.1	100	XM_023083737.1	100	XM_023073596.1	100	XM_023081963.1	100
									XM_023091092.1	100

	LCYB		CYP97A		BCH		VDE		ZEP		NXS	
	Gene ID	FC (%)	Gene ID	FC (%)	Gene ID	FC (%)	Gene ID	FC (%)	Gene ID	FC (%)	Gene ID	FC (%)
Malus domestica	MD00G1049000	100	MD04G1239800	100	MD01G1208300	100	MD12G1255200	100	MD02G1172400	100	MD06G1045700	100
			MD12G1257000	100	MD07G1278900	100			MD15G1284500	100	MD00G1132400	100
					MD06G1089200	100					MD09G1253000	100
Fragaria vesca	XM_004303559.1	100	XM_004302087.1	100	XM_004308006.1	100	XM_004302077.1	100	XM_004306336.1	93.3	XM_004300264.1	100
					XM_004300765.1	100						
Ulmus minor	Umino49479.1	100	Umino30099.1	100	Umino13563.1	100	Umino30042.1	100	Umino38793.1	100	Umino04414.1	100
					Umino44146.1	100						
Hippophae rhamnoides	Hiprha1gene27597	100	Hiprha1gene26199	100	Hiprha1gene05693	100	Hiprha1gene26091	100	Hiprha1gene15592	100	Hiprha1gene22818	100
											Hiprha1gene00108	100
Quercus robur	QR_050389511.1	100	QR_050386579.1	100	QR_050397113.1	100	QR_050386520.1	100	QR_050435776.1	100	QR_050413833.1	100
			QR_050386575.1	100	QR_050410144.1	100	QR_050386525.1	100				
			QR_050386572.1	100			QR_050386529.1	100				
Castanea mollissima	Cm_g45431.t1	100	Cm_g3450.t1	100	Cm_g34297.t1	100	Cm_g2015.t1	100	Cm_g19348.t1	100	Cm_g40138.t2	100
	Cm_g36967.t1	100			Cm_g35921.t1	100					Cm_g40138.t1	100
Juglans regia	JR_018961643.2	100	JR_018962194.2	100	JR_018954124.2	100	JR_018971416.2	100	JR_018989429.2	100	JR_018959943.2	100
					JR_018983068.2	100						
					JR_018997000.2	100						
Carya illinoinensis	XM_043086101.1	100	XM_043132643.1	100	XM_043126582.1	100	XM_043131704.1	100	XM_043118141.1	100	XM_043121614.1	100
					XM_043129314.1	100	XM_043131705.1	100				
					XM_043091779.1	100						
***Gynostemma pentaphyllum*2**	GP14085.1	100	GP36538.1	100	GP35469.1	100	GP11374.1	100	GP39143.1	100	GP15472.1	100
					GP20560.1	100			GP39222.1	100	GP14998.1	100
***Momordica charantia*2**	Moc01g17190.1	100	Moc10g06010.1	100	Moc10g01250.1	100	Moc08g38440.1	100	Moc02g13110.1	100	Moc08g12330.1	100
					Moc06g38230.1	100						
***Luffa aegyptiaca*1**	Lcy04g005840.1	100	Lcy11g010510.1	100	Lcy11g001300.1	100	Lcy08g010620.1	100	Lcy07g004710.1	100	Lcy02g001400.1	100
					Lcy02g013470.1	100						
***Cucumis sativus*1**	CS_004150713.3	100	CS_004133705.3	100	CS_004140710.3	100	NM_001305728.1	100	NM_001305784.1	100	CS_004145812.3	100
					CS_004143925.3	100					CS_031882497.1	100
											CS_031882498.1	100
***Citrullus lanatus*2**	Cla97C04G070940.1	100	Cla97C10G191610.1	100	Cla97C05G090480.1	100	Cla97C11G216330.1	100	Cla97C02G038200.1	100	Cla97C05G093720.2	100
					Cla97C01G002480.2	100						
***Benincasa hispida*1**	BH_039026592.1	100	BH_039050399.1	100	BH_039021520.1	100	BH_039034565.1	100	BH_039045392.1	100	BH_039031262.1	100
					BH_039051224.1	100						
***Cucurbita pepo*1**	CP_023689700.1	100	CP_023697021.1	100	CP_023696049.1	100	CP_023681009.1	100	CP_023677552.1	100	CP_023671875.1	100
	CP_023661496.1	100			CP_023685715.1	100					CP_023671874.1	100
					CP_023695199.1	100						
					CP_023695198.1	100						
***Cucurbita moschata*1**	XM_023077230.1	100	XM_023073576.1	100	XM_023089097.1	100	XM_023081828.1	100	XM_023091781.1	100	XM_023079271.1	100
	XM_023069390.1	100			XM_023066853.1	100			XM_023091780.1	100	XM_023079268.1	100
					XM_023073255.1	100					XM_023079270.1	100

Table S5: Genes used for expression analyses of flavonoid biosynthesis genes in outgroups and Cucurbitaceae

For each species, gene IDs corresponding to flavonoid biosynthesis enzymes are listed. Functional conservation (FC, %) indicates the percentage of conserved amino acid residues required for the annotated enzyme function. '---' indicates gene sequence not detected.

	CHS		***CHI***		***F3H***		***F3'H***		***FLS***		***DFR***		***ANS***		***arGST***		***LAR***		***ANR***	
	Gene ID	FC (%)	Gene ID	FC (%)	Gene ID	FC (%)	Gene ID	FC (%)	Gene ID	FC (%)	Gene ID	FC (%)	Gene ID	FC (%)	Gene ID	FC (%)	Gene ID	FC (%)	Gene ID	FC (%)
Malus domestica	MD04G1003400	100	MD01G1118000	100	MD15G1246200	100	MD06G1201700	100	MD08G1168600	100	MD15G1024100	100	MD03G1001100	100	MD17G1272100	100	MD16G1048500	100	MD05G1335600	100
	MD04G1003300	100	MD01G1117800	100	MD02G1132200	100	MD14G1210700	100	MD08G1121600	100	MD08G1028600	100	MD06G1071600	100			MD13G1046900	100		100
	MD04G1003000	100	MD01G1118100					100	MD15G1354100	100							MD06G1211400	100		
	MD13G1285100	100														100				
Fragaria vesca	XM_004306495.1	100	XM_004307403.1	100	XM_004287766.1	100	XM_004299260.1	100	XM_004290789.1	100	XM_004291810.1	100	XM_004298672.1	100	XM_004288530.1	100	XM_004297096.1	100	XM_004309614.1	100
	XM_004306494.1	100																		
Ulmus minor	Umino03942.1	100	Umino11916.1	100	Umino37973.1	100	Umino46177.1	100	Umino30799.1	100	Umino05936.1	100	Umino44418.1	100	Umino31194.1	100	Umino36433.1	100	Umino00737.2	100
	Umino52268.1	100			Umino12489.1	100														
Hippophae rhamnoides	Hiprha1gene04371	100	Hiprha1gene02313	100	Hiprha1gene24025	100	Hiprha1gene19656	100	Hiprha1gene19276	100	Hiprha1gene25674	100	Hiprha1gene06498	100	---		Hiprha1gene24235	100	Hiprha1gene02920	100
	Hiprha1gene30592	100			Hiprha1gene29544	100			Hiprha1gene04820	100	Hiprha1gene11285	100					Hiprha1gene07946	100		
	Hiprha1gene15431	100															Hiprha1gene05323	100		
	Hiprha1gene17498	100																		
Quercus robur	QR_050414649.1	100	QR_050394358.1	100	QR_050436431.1	100	QR_050400788.1	100	QR_050401176.1	100	QR_050411544.1	100	QR_050408671.1	100	QR_050414417.1	100	QR_050385978.1	100	QR_050432874.1	100
	QR_050383154.1	100					QR_050400789.1	100			QR_050411541.1	100								
							QR_050400791.1	100			QR_050411542.1	100								
							QR_050400792.1	100												
							QR_050400790.1	100												
Castanea mollissima	Cm_g41904.t1	100	Cm_g26679.t1	100	Cm_g42151.t1	100	---	100	Cm_g26539.t1	100	Cm_g10343.t1	100	Cm_g18792.t1	100	Cm_g44678.t1	100	Cm_g41413.t1	95	Cm_g40265.t1	100
	Cm_g6534.t1											100				100				
Juglans regia	JR_018966498.2	100	JR_018972487.2	100	JR_018974079.2	100	JR_018954394.2	100	JR_018965395.2	100	JR_018965609.2	100	JR_018991659.2	100	JR_018954616.2	100	JR_035690843.1	100	JR_018950001.2	100
	JR_018971548.2	100							JR_035695146.1	100							JR_035691108.1	100		
	JR_018961711.2	100							JR_018953238.2	100							JR_018980541.2	100		
									JR_018953269.2	100										
									JR_018961611.2	100										
Carya illinoinensis	XM_043130600.1	100	XM_043097661.1	100	XM_043132107.1	100	XM_043093388.1	100	XM_043122765.1	100	XM_043133112.1	100	XM_043093893.1	100	XM_043120910.1	100	XM_043104692.1	100	XM_043087336.1	100
	XM_043132918.1	100							XM_043122900.1	100	XM_043133113.1	100					XM_043109036.1	100		
	XM_043110823.1	100							XM_043118318.1	100										
									XM_043119140.1	100										
									XM_043122444.1	100										
Gynostemma pentaphyllum2	GP15028.1	100	GP02930.1	100	GP36709.1	100	GP21533.1	97	GP02556.1	100	GP16318.2	89	---		---		---		---	
	GP06757.1	100							GP29425.1	100	GP16318.1	89								
	GP06947.1	100																		
Momordica charantia2	Moc08g12970.1	100	Moc04g34280.1	95	Moc10g29680.1	100	Moc06g02700.1	83	Moc04g31040.1	100	---		---		---		---		---	
Luffa aegyptiaca1	Lcy09g021280.1	100	Lcy09g005850.1	95	Lcy11g020370.1	100	Lcy03g015720.1	100	Lcy09g011830.1	100	---		---		---		---		---	
Cucumis sativus1	CS_004145659.3	100	CS_004149769.3	95	CS_004146092.3	92	CS_004138144.3	100	CS_004140814.3	100	---		---		---		---		---	
					CS_004140448.3	92														
Citrullus lanatus2	Cla97C05G094290.1	100	Cla97C09G181670.1	95	Cla97C10G185310.1	100	Cla97C06G125240.1	100	Cla97C08G148220.1	100	---		---		---		---		---	
Benincasa hispida1	BH_039036415.1	100	BH_039034903.1	90	BH_039020680.1	100	BH_039022770.1	100	BH_039029707.1	100	---		---		---		---		---	
					BH_039040061.1	100														
Cucurbita pepo1	CP_023672485.1	100	CP_023693934.1	95	CP_023696035.1	92	CP_023673727.1	100	CP_023695380.1	100	---		---		---		---		---	
			CP_023693933.1		CP_023684146.1	92	CP_023673726.1	100	CP_023660628.1	100										
Cucurbita moschata1	XM_023078567.1	100	XM_023082756.1	95	XM_023067406.1	100	XM_023077843.1	100	XM_023108724.1	100	---		---		---		---		---	
									XM_023108732.1	100										
									XM_023082614.1	100										

References

Chomicki, G. and Renner, S.S. (2015) Watermelon origin solved with molecular phylogenetics including Linnaean material: another example of museomics. *New Phytologist*, **205**, 526–532.

Czechowski, T., Stitt, M., Altmann, T., Udvardi, M.K. and Scheible, W.-R. (2005) Genome-Wide Identification and Testing of Superior Reference Genes for Transcript Normalization in Arabidopsis. *Plant Physiol*, **139**, 5–17.

Ferreira, M.J., Silva, J., Pinto, S.C., Coimbra, S., Ferreira, M.J., Silva, J., Pinto, S.C. and Coimbra, S. (2023) I Choose You: Selecting Accurate Reference Genes for qPCR Expression Analysis in Reproductive Tissues in Arabidopsis thaliana. *Biomolecules*, **13**.

Hong, S.M., Bahn, S.C., Lyu, A., Jung, H.S. and Ahn, J.H. (2010) Identification and Testing of Superior Reference Genes for a Starting Pool of Transcript Normalization in Arabidopsis. *Plant Cell Physiol*, **51**, 1694–1706.

Anon Plants of the World Online | Kew Science. *Plants of the World Online*. Available at: https://powo.science.kew.org/

Schaefer, H. and Renner, S.S. (2011) Phylogenetic relationships in the order Cucurbitales and a new classification of the gourd family (Cucurbitaceae). *TAXON*, **60**, 122–138.

Souček, P., Pavlů, J., Medveďová, Z., Reinöhl, V. and Brzobohatý, B. (2017) Stability of housekeeping gene expression in Arabidopsis thaliana seedlings under differing macronutrient and hormonal conditions. *J. Plant Biochem. Biotechnol.*, **26**, 415–424.
